# Swine Enteric Colibacillosis in Spain: Pathogenic Potential of *mcr-1* ST10 and ST131 *E. coli* Isolates

**DOI:** 10.3389/fmicb.2018.02659

**Published:** 2018-11-05

**Authors:** Isidro García-Meniño, Vanesa García, Azucena Mora, Dafne Díaz-Jiménez, Saskia C. Flament-Simon, María Pilar Alonso, Jesús E. Blanco, Miguel Blanco, Jorge Blanco

**Affiliations:** ^1^Laboratorio de Referencia de Escherichia coli (LREC), Departamento de Microbioloxía e Parasitoloxía, Facultade de Veterinaria, Universidade de Santiago de Compostela (USC), Lugo, Spain; ^2^Unidad de Microbiología, Hospital Universitario Lucus Augusti (HULA), Lugo, Spain

**Keywords:** *E. coli*, ST10, ST131, *mcr-1*, EPEC, ETEC, STEC, post-weaning diarrhea

## Abstract

This is a wide epidemiological study of 499 *E. coli* isolates recovered from 179 outbreaks of enteric colibacillosis from pig production farms in Spain during a period of 10 years. Most samples were of diarrheagenic cases occurred during the post-wean period (PWD) which showed to be significantly associated with ETEC (67%) followed by aEPEC (21.7%). On the contrary, aEPEC was more prevalent (60.3%) among diarrheas of suckling piglets, followed by ETEC (38.8%). STEC/ETEC or STEC were recovered in 11.3 and 0.9% of PWD and neonatal diarrhea, respectively. Detection of the F4 colonization factor was not significantly different between isolates recovered from neonatal pigs and those recovered post wean (40.5 versus 27.7%) while F18 was only present among PWD isolates (51.5% of ETEC, STEC, and STEC/ETEC isolates). We also found a high prevalence of resistance to colistin related to the presence of the *mcr-1* gene (25.6% of the diarreagenic isolates). The characterization of 65 representative *mcr-1* isolates showed that all were phenotypically resistant to colistin (>2 μg/ml), and most (61 of 65) multidrug-resistant (MDR). Six ETEC and one STEC *mcr-1* isolates were also carriers of ESBL genes. In addition, other seven *mcr-1* isolates harbored *mcr-4* (three ETEC) and *mcr-5* (two ETEC and two aEPEC) genes. In the phylogenetic analysis of the 65 *mcr-1* diarrheagenic isolates we found that more than 50% (38 out of 65) belonged to A-ST10 Cplx and from those, 29 isolates showed the clonotype CH11-24. In this study, we also recovered 18 ST131 isolates including seven *mcr-1* carriers. To the best of our knowledge, this would be the first report of ST131 *mcr-1* isolation in pigs. Worryingly, the swine *mcr-1* ST131 carriers also showed MDR, including to trimethoprim-sulfamethoxazole, tobramycin, gentamicin and ciprofloxacin. In the PFGE-macrorestriction comparison of clinical swine and human ST131, we found high similarities (≥85%) between two pig and two human ST131 isolates of virotype D5. Acquisition of *mcr-1* by this specific clone means an increased risk due to its special feature of congregating virulence and resistance traits, together with its spread capability. Here we show a potential zoonotic swine source of ST131.

## Introduction

Neonatal and post-weaning diarrheal (PWD) disease affecting pigs during the first weeks after birth result in significant economic losses for the pig industry due to mortality, decreased weight gain, costs derived of treatment and handling, and vaccinations (Fairbrother et al., 2005; Luppi, 2017).

Among the etiologic agents, there are certain *Escherichia coli* pathotypes commonly implicated in enteric diseases in piglets, namely, Shiga toxin-producing *E. coli* (STEC) and enterotoxigenic *E. coli* (ETEC). STEC isolates carrying the shiga toxin type 2e (Stx2e) are the causative agents of edema disease in weaning piglets, while ETEC isolates encoding the heat stable (STa, STb) and/or heat labile (LT) enterotoxins cause secretory diarrhea in newborn and weaned piglets (Nagy et al., 1999; Fairbrother et al., 2005; Fairbrother and Gyles, 2012). Furthermore, some isolates harbor both the Stx2e and enterotoxin genes, being able to cause symptoms of edema disease and diarrhea in the same animal (STEC/ETEC) (Barth et al., 2011). A common feature of STEC and/or ETEC swine pathotypes is the expression of specific fimbrial adhesins which allow bacterial colonization of the mucosal surface. The most commonly reported are of types F4 (previously known as K88) and F18 (F107, 2134P, and 8813), both with different antigenic variants: three for F4 (ab, ac, and ad), with F4ac being the most prevalent, and two main variants for F18 (F18ab associated with edema disease and F18ac with PWD) (Westerman et al., 1988; Rippinger et al., 1995; Francis, 2002). Other associated fimbriae of lower prevalence include F5 (K99), F6 (987P), and F41, whose number of active receptors present on the intestinal epithelial cells decreases with age (Vu Khac et al., 2006; Luppi et al., 2016). Enteropathogenic *E. coli* (EPEC) is another pathotype found in pigs with enteric colibacillosis (Brand et al., 2017; Malik et al., 2017) which was first associated with diarrhea in infants (Shuman and Stock, 1956). EPEC isolates possess an outer membrane protein adhesin or intimin (Eae), responsible for intimate attachment of the bacteria to the host intestinal epithelium, which together with a complex secretion system leads to the development of the “attaching and effacing” (AE) lesion (Frankel and Phillips, 2008).

Colistin (polymyxin E) is one of the few cationic antimicrobial peptides commercialized in both human and veterinary medicine. In humans, colistin is used as a last resort against infections of multidrug-resistant (MDR) Gram-negative bacteria (Poirel et al., 2017). However, it has been extensively used since the 1960s in food animals, and particularly in swine with different purposes: therapeutically, prophylactically, and even for growth promotion (Rhouma et al., 2016). Since the description of the plasmid-mediated colistin resistance *mcr-1* gene in late 2015 (Liu et al., 2016), several *mcr* genes have been described (Xavier et al., 2016; Borowiak et al., 2017; Carattoli et al., 2017; Liu et al., 2017; García et al., 2018; Hammerl et al., 2018; Rebelo et al., 2018). Livestock, and particularly pig farming, has been singled out as the principal reservoir for colistin resistance spread (Rhouma et al., 2016). Besides, co-occurrence on the same plasmid of *mcr* and extended-spectrum beta-lactamase (ESBL) or other beta-lactamase genes (such as carbapenemase) are being increasingly identified in isolates from different origins (Bai et al., 2016; Haenni et al., 2016). In addition, one of the major health concerns associated with *E. coli* is the role of certain clonal groups in the emergence and dissemination of antimicrobial resistance, namely those belonging to sequence type (ST) 10, ST69, ST131, ST405, ST410, or ST648 (Hansen et al., 2014; Falgenhauer et al., 2016; Johnson et al., 2017). Of particular importance is the ST131 due to its prevalence in community- and hospital-acquired urinary tract infections (UTIs) (Nicolas-Chanoine et al., 2014; Kallonen et al., 2017), and whose potential contribution to the global dissemination of antimicrobial resistances has been also highlighted in food-producing animals (Mora et al., 2010).

In the present study, we characterized a collection of *E. coli* isolates obtained in Spain during the period 2006–2016 from pigs suffering enteric colibacillosis with three main aims: (i) to define clonal groups of clinical importance in swine enteric colibacillosis; (ii) to analyze the rates of antibiotic resistance in pig farming in Spain, including mobile resistance to colistin (*mcr* gene); and (iii) to gain knowledge about the presence and zoonotic potential of ST131 isolates of livestock origin.

## Materials and Methods

### *E. coli* Collection

A total of 464 rectal samples from pigs suffering diarrhea were tested for routine diagnosis of enteric colibacillosis at the Reference Laboratory of *Escherichia coli* (LREC), in Lugo, Spain. The samples were collected in farms from different Spanish regions between 2006 and 2016, mainly of diarrheas after weaning (73%) and the remaining (27%) from suckling piglets.

Swabs were plated on lactose MacConkey agar (LMAC) and sorbitol MacConkey agar (Oxoid) supplemented with cefixime (0.05 mg/l) and potassium tellurite (2.5 mg/l) (CTSMAC), and incubated at 37°C for 18–24 h. Afterward, the confluent growth of all plates were tested to detect the presence of ETEC, STEC, and EPEC by PCR based on specific genes encoding toxins (LT, STa, STb, Stx1, Stx2, Stx2e, and HlyA), fimbriae (F4, F5, F6, F18, and F41), intimin (Eae), and bundle-forming pilus (BFP) (Blanco et al., 2006; Vu Khac et al., 2006; Vu Khac et al., 2007; Supplementary Table S1). Confluents were also screened for *rfb*O25 using specific primers described by Clermont et al. (2008) to presumptively detect the ST131 clonal group. For each PCR-positive culture, five *E. coli*-like colonies from LMAC and/or CTSMAC plates were plated on tryptone soy agar (Oxoid) and individually analyzed by PCR. Those colonies showing different genetic characteristics for the selected targets were stored at room temperature in nutrient broth (Difco^TM^) with 0.75% nutrient agar (Difco^TM^) for further characterization. In total, 499 *E. coli* isolates were examined in this study representing 179 diarrheagenic outbreaks.

### O and H Typing

Determination of O:H antigens was carried out following the method described by Guinée et al. (1981) with O1 to O185 and H1 to H56 antisera, respectively. Isolates that did not react with any O antisera were classified as non-typeable (ONT), and non-motile isolates (HNM) were further analyzed by PCR to determine their flagellar genes (Mora et al., 2018; Supplementary Table S2).

### Detection of *mcr-1* and Other *mcr* Genes

The 499 *E. coli* isolates were investigated for the presence of *mcr-1* gene by PCR as detailed elsewhere (Liu et al., 2016). From the positive *mcr-1* isolates, a representative group displaying different serotypes/pathotypes was further characterized as described below, including the screening of *mcr-2*, *3*, *4*, and *5* using primers and conditions of previous studies (Xavier et al., 2016; Borowiak et al., 2017; Carattoli et al., 2017; Yin et al., 2017; Supplementary Table S3).

### Antimicrobial Susceptibility and Genotypic Characterization of β-Lactamases

Antimicrobial susceptibility was determined by minimal inhibitory concentrations (MICs) using the MicroScan WalkAway^®^-automated system (Siemens Healthcare Diagnostics, CA, United States) according to the manufacturer’s instructions. The antibiotics tested included ticarcillin, aztreonam, ceftazidime, cefepime, ampicillin-sulbactam, piperacillin-tazobactam, imipenem, meropenem, amikacin, gentamicin, tobramycin, levofloxacin, ciprofloxacin, trimethoprim-sulfamethoxazole, fosfomycin, colistin, minocycline, and tigecycline. Additionally, resistance to ampicillin, cefotaxime, chloramphenicol, and nalidixic acid was determined by disk (Becton Dickinson, Sparks, MD, United States) diffusion assays. All results were interpreted according to the CLSI (CLSI, 2017). Genetic identification of the ESBLs was performed by PCR using the TEM, SHV, CTX-M-1, and CTX-M-9 group-specific primers followed by amplicon sequencing (Mora et al., 2013; Supplementary Table S3).

### Phylogroups, Clonotypes, and Sequence Types (STs)

The phylogenetic relatedness of the isolates was analyzed on the basis of their phylogroups, clonotypes, and STs. The assignment to the main *E. coli* phylogenetic groups (A, B1, B2, C, D, E, and F) was performed using the quadruplex phylogroup assignment method described by Clermont et al. (2013) based on the presence/absence of the four genetic targets *arpA*, *chuA*, *yjaA*, and TspE4.C2 (Supplementary Table S4). MLST was performed following the Achtman seven-locus scheme. Briefly, internal fragments of seven housekeeping genes (*adk*, *fumC*, *gyrB*, *icd*, *mdh*, *purA*, and *recA*) were amplified and sequenced using published criteria and primers (Wirth et al., 2006; Supplementary Table S4). The allelic profile for each isolate was determined through the Enterobase website.^1^ The clonotyping was based on the internal 469-nucleotide (nt) and 489-nt sequence of the *fumC* and *fimH* genes, respectively (Weissman et al., 2012; Supplementary Table S4). Allele assignments for *fimH* were determined using the fimtyper database available at the Center for Genomic Epidemiology website.^2^ The combination of *fumC* (allele obtained from MLST) and *fimH* allele designations was used as the CH “type.” Finally, a neighbor-joining tree was constructed by MEGA6 (Tamura et al., 2013) with the concatenated sequences of the seven housekeeping genes to confirm the consistency of the phylogroup assignations.

### Characterization of ST131 Isolates

Isolates confirmed as ST131 by MLST were additionally investigated for extraintestinal virulence markers. Based on the results, the isolates were considered to conform the extraintestinal pathogenic *E. coli* (ExPEC) status if positive for two or more of five markers, including *papAH* and/or *papC*, *sfa/focDE*, *afa/draBC*, *kpsM II*, and *iutA* (Johnson et al., 2003), and the uropathogenic (UPEC) status if positive for three or more of four markers, including *chuA*, *fyuA*, *vat*, and *yfcV* (Spurbeck et al., 2012; Supplementary Table S1). The virotype of the ST131 isolates was established according to the scheme described by Dahbi et al. (2014) based on the presence or absence of certain extraintestinal virulence genes (*afa/draBC*, *afa* operon FM955459, *iroN*, *sat*, *ibeA*, *papG II*, *papG III*, *cnf1*, *hlyA*, *cdtB*, *neuC-K*, *kpsM II-K2*, and *kpsM II-K5*).

### Pulsed Field Gel Electrophoresis (PFGE)

The similarity within the *E. coli* clonal group ST131 was established comparing the *XbaI*-PFGE profiles of the isolates which were obtained following the PulseNet protocol,^3^ and imported into BioNumerics (Applied Maths, St-Martens-Latern Belgium) to perform a dendrogram with the UPGMA algorithm based on the Dice similarity coefficient and applying 1% of tolerance in the band position.

### Statistical Analysis

Differences were compared by a two-tailed Fisher’s exact test. *P* values <0.05 were considered statistically significant.

## Results

### Diarrheagenic Pathotypes and Serogroups

The 499 *E. coli* isolates analyzed in this work had been obtained from 464 fecal samples of 179 diarrheagenic outbreaks occurred in different geographic areas of Spain. By PCR, 481 of those 499 isolates shown diarrheagenic pathotypes, while 18 isolates recovered by means of the *rfb*O25 screening and negative for the enteric virulence genes were later investigated concerning the ST131 clonal group.

Specifically, the major pathotypes found among the 481 diarrheagenic isolates were ETEC (277 isolates; 57.6%) positive for genes encoding enterotoxins (*eltA*, and/or *estA*, and/or *estB*) and aEPEC (156 isolates; 32.4%) positive for *eae* but negative for *bfpA* (therefore classified as atypical EPEC, aEPEC). The remaining isolates were assigned as STEC/ETEC (33 isolates; 6.9%) positive for both Stx2e and enterotoxin-encoding genes (*stx*_2e_ and *estB* and/or *estA*) and STEC (15 isolates; 3.1%) positive for *stx*_2e_. According to the age of the affected animals, aEPEC was the most prevalent pathotype in suckling piglets (60.3%) followed by ETEC (38.8%). By contrast, ETEC isolates were the most prevalent in PWD (67.0%) followed by aEPEC (21.7%) (*P* < 0.001 for both comparisons). Furthermore, STEC/ETEC were recovered in 9.0% of PWD versus 0.9% of neonatal diarrheas (*P* < 0.005); and STEC only in piglets after weaning (2.3%). The colonization factors identified among ETEC isolated from neonatal diarrhea were F4, F5 + F41, and F6 (40.5, 16.7, and 11.9%, respectively); while for PWD no isolates were positive for F5, F6, or F41; however F4 was detected at a level not significantly different to neonatal diarrhea isolates (27.7% of PWD ETEC isolates; *P* > 0.05). Finally, F18 was only detected in PWD (51.5%) including 45.5% of ETEC, 82.1% of STEC/ETEC, and 80.0% of STEC isolates.

Among the 481 diarrheagenic isolates, a total of 50 different serogroups were distinguished by serotyping. Also, 114 (23.7%) isolates that did not react with any of the O1 to O185 antisera were classified as non-typeable (ONT). In spite of this variability, we found a significant association between pathotypes and certain serogroups. Thus, O108, O138, O141, O149, and O157 accounted for 75.1% of ETEC; O26, O49, O80, and O111 for 39.1% of aEPEC; O138 and O141 for 57.6% of STEC/ETEC; and O139 accounted for 46.7% of STEC (Table 1).

**Table 1:** Association between serogroups, pathotypes, and presence of *mcr-1* gene among the 481 *E. coli* isolates involved in swine colibacillosis (Spain, 2006–2016).

Serogroup^a^	ETEC *N* = 277 No. isolates (%)	aEPEC *N* = 156 No. isolates (%)	STEC/ETEC *N* = 33 No. isolates (%)	STEC *N* = 15 No. isolates (%)	*mcr-1 N* = 123 No. isolates (%)
O2	1 (0.4)	6 (3.8)	–	2 (13.3)	**8 (6.5)**
O4	–	2 (1.3)	–	–	–
O5	1 (0.4)	1 (0.6)	–	–	1(0.8)
O7	3 (1.1)	–	–	–	2 (1.6)
O8	4 (1.4)	–	–	–	1 (0.8)
O14	1 (0.4)	–	–	–	–
O15	7 (2.5)	5 (3.2)	–	–	**5 (4.1)**
O20	4 (1.4)	1 (0.6)	–	–	–
O22	–	–	–	1 (6.7)	–
O26	–	**17 (10.9)**	–	–	**7 (5.7)**
O28	–	1 (0.6)	–	–	–
O35	5 (1.8)	–	–	–	2 (1.6)
O36				1 (6.7)	1(0.8)
O39	–	1 (0.6)	–	–	–
O45	9 (3.2)	2 (1.3)	–	–	**6 (4.9)**
O49	–	**21 (13.5)**	–	–	–
O51	–	2 (1.3)	–	–	1 (0.8)
O55	–	1 (0.6)	–	–	–
O65	–	–	1 (3)	–	–
O76	–	6 (3.9)	–	–	–
O80	–	**12 (7.7)**	–	–	–
O81		1 (0.6)			1(0.8)
O86	–	–	1 (3)	–	–
O88	1 (0.4)	1 (0.6)	–	–	1(0.8)
O98	2 (0.7)	–	–	–	–
O101	5 (1.8)	–	–	–	–
O103	–	5 (3.2)	–	–	2 (1.6)
O108	**43 (15.5)**	3 (1.9)	–	–	–
O111	–	**11 (7.0)**	–	–	**5 (4.1)**
O115	–	2 (1.3)	–	–	–
O118	1 (0.4)	1 (0.6)	–	–	1 (0.8)
O123	–	5 (3.2)	–	–	3 (2.4)
O127	–	2 (1.3)	–	–	–
O137	1 (0.4)	–	–	–	–
O138	**15 (5.4)**	–	**8 (24.2)**	–	**10 (8.1)**
O139	–	–	–	**7 (46.7)**	1 (0.8)
O141	**18 (6.5)**	–	**11 (33.3)**	–	**26 (21.1)**
O142	–	–	–	1 (6.7)	–
O145	–	6 (3.9)	–	–	2 (1.6)
O147	2 (0.7)	–	–	–	–
O149	**14 (5.1)**	–	–	–	–
O153	–	7 (4.5)	–	–	–
O157	**57 (20.6)**	7 (4.5)	1 (3)	–	**26 (21.1)**
O158	–	–	–	1 (6.7)	–
O159	1(0.4)	–	1 (3)	–	1 (0.8)
O163	1 (0.4)	–	–	–	–
O169	1 (0.4)	–	–	–	–
O174	–	1 (0.6)	–	–	–
O177	–	3 (1.9)	–	–	1 (0.8)
O180	–	1 (0.6)	–	–	–
ONT	80 (28.9)	22 (14.1)	10 (30.3)	2 (13.3)	9 (7.3)

*^a^Isolates that did not react with any O antisera were classified as non-typeable (ONT).*

### Prevalence of *mcr-1* Among Diarrheagenic Isolates

A total of 123 (25.6%) out of 481 diarreagenic isolates harbored the *mcr-1* gene, without significant differences in relation to their pathotyes (71 ETEC, 36 aEPEC, 10 STEC/ETEC, and 6 STEC isolates) (*P* > 0.05 for all comparisons); nevertheless, there was a significant association with the age of the affected animals (33.5% of PWD isolates versus 8.7% of neonatal diarrhea) (*P* < 0.001). The 123 isolates showed 23 serogroups, although 75.6% belonged to only 8 (O2, O15, O26, O45, O111, O138, O141, and O157) (Table 1).

### Serotypes, Antimicrobial Resistances, ESBL Types, and *mcr* Genes

From the 123 *mcr-1 E. coli* involved in swine colibacillosis, a representative group of 65 isolates was selected for further characterization. The selection included approximately 50% of each pathotype (33 out of 71 ETEC, 24 out of 36 aEPEC, 5 out of 10 STEC/ETEC, and 3 out of 6 STEC), as well as the serogroups representing 75.6% of the *mcr-1* isolates (O2, O15, O26, O45, O111, O138, O141, and O157) together with other less prevalent (O7, O8, O35, O51, O103, O118, O123, O139, O145, and O177).

By serotyping, the 33 ETEC *mcr-1* isolates showed 11 different O:H combinations being O157:HNM, O141:H4, and O138:H14 the most prevalent (12, seven, and three isolates, respectively); the five STEC/ETEC isolates were O141:H4; and the three STEC showed three different serotypes (O2:HNM, O139:H1, and O141:HNM) (Table 2). Finally, the 24 aEPEC *mcr-1* isolates belonged to 12 serotypes, with O26:H11, O2:H40, and O123:H11 as the most prevalent (five, four, and three isolates, respectively) (Table 3).

**Table 2:** Molecular characterization of the 41 ETEC, STEC, and STEC/ETEC *mcr-1*-positive isolates.

Pathotype (No. isolates)	Serotype^a^-PG-ST	CH^b^	No. isolates	ESBL typing (No. isolates)	Virulence gene profile (No. isolates)	Resistance profile^c^ (isolates with *mcr* co-occurrence and type)
ETEC (33)	O141:H4-A-ST10	11-24	2	–	STa, STb, F18ac, HlyA (1)	AMP AMP/SAM CHL COL GEN TI TMP/SMX TOB
					STa, STb (1)	AMP AMP/SAM CHL COL GEN TI TMP/SMX TOB
	O157:HNM-A-ST10	11-24	10	CTX-M-14 (3)	LT, STb, K88ac, HlyA (5)	AMP AMP/SAM ATM COL CTX FEP MI NAL TI
						AMP AMP/SAM CHL COL CTX FEP MI NAL TI TMP/SMX
						AMP AMP/SAM COL CTX FEP GEN MI NAL TI TMP/SMX TOB
						AMP AMP/SAM COL GEN MI NAL TI TMP/SMX TOB
						CIP COL LEV MI NAL
					LT, STb, K88ac (2)	AMP AMP/SAM COL GEN MI NAL TI TMP/SMX TOB **(*mcr-1*/*mcr-5*)**
						COL MI NAL TMP/SMX
					LT, STb, HlyA (1)	AMP COL GEN MI NAL TI TMP/SMX TOB **(*mcr-1*/*mcr-5*)**
					LT, K88ac, HlyA (1)	AMP AMP/SAM COL GEN MI NAL TI TMP/SMX TOB
					LT, STb, K88ac, HlyA (1)	AMP COL GEN MI NAL TI TMP/SMX TOB
		11-0	1		LT, STb, K88ac, HlyA (1)	AMP AMP/SAM COL GEN NAL TI TMP/SMX TOB
	O45:HNM-A-ST10	11-24	1	–	LT, F18ac, HlyA (1)	AMP AMP/SAM CIP COL GEN LEV NAL TI TMP/SMX TOB **(*mcr-1*/*mcr-4*)**
	ONT:HNM-A-ST10	11-24	1	–	LT, F18ac, HlyA (1)	AMP AMP/SAM CIP COL GEN LEV NAL TI TMP/SMX TOB **(*mcr-1*/*mcr-4*)**
		11-94	2	CTX-M-14 (1)	STa, STb (1)	AMP AMP/SAM CHL COL GEN NAL TI TMP/SMX TOB
					STa, STb, F18ac, HlyA (1)	AMP AMP/SAM ATM CHL CIP COL CTX FEP GEN LEV NAL TI TMP/SMX TOB
	O35:H6-A-ST10	11-45	1	–	STb (1)	AMP COL NAL TI **(*mcr-1*/*mcr-4*)**
	O141:H4-A-ST5786	11-24	5	CTX-M-14 (1)	STa, STb, F18ac, HlyA (2)	AMP COL TMP/SMX
						COL FOS MI TMP/SMX NAL
					STa, F18ac, HlyA (1)	AMP AMP/SAM ATM CHL COL CTX FEP MI NAL TI
					STa, STb, F18ac, HlyA (1)	AMP CHL COL GEN NAL TI TMP/SMX TOB
					STa, STb, F18ac, HlyA (1)	CHL COL FOS TMP/SMX
	O7:H4-A-ST93	11-27	1	SHV-12 (1)	STb (1)	AMP AMP/SAM ATM CAZ CHL COL CTX TI
	O138:H10-A-ST100	27-0	2	–	LT, STb, K88ac, HlyA (1)	AMP AMP/SAM COL GEN MI NAL TI TOB
					STb, K88ac, HlyA (1)	COL MI TMP/SMX
	O8:HNM-A-ST398	7-171	1	–	STb (1)	CHL COL
	O157:HNM-B1-ST156	29-38	1	–	STb (1)	AMP AMP/SAM CHL CIP COL LEV MI NAL TI TMP/SMX TOB
	O138:H14-E-ST42	28-65	3	–	LT, STa, STb, F18ac, HlyA (1)	AMP AMP/SAM CIP COL ^∗^FOS LEV MI NAL TI
					LT, STb, F18ac, HlyA (1)	AMP AMP/SAM CHL COL ^∗^FOS TI TMP/SMX NAL
					LT, STa, STb, F18ac, HlyA (1)	CHL COL ^∗^FOS GEN TOB NAL
	O15:H45-E-ST118	4-331	1	–	STb (1)	AMP AMP/SAM CHL COL GEN MI NAL TI TOB
	O45:H45-E-ST4247	550-400	1	–	STb (1)	COL NAL
STEC/ETEC (5)	O141:H4-A-ST10	11-24	5	–	STa, STb, Stx2e, F18ac, HlyA (3)	AMP AMP/SAM CHL COL ^∗^FOS GEN TI TMP/SMX TOB (2)
						AMP AMP/SAM CHL COL TI TMP/SMX
					STa, STb, Stx2e, F18ac HlyA (2)	AMP AMP/SAM CHL COL GEN TI TMP/SMX TOB (2)
STEC (3)	O2:HNM-A-ST10	11-23	1	–	Stx2e, HlyA (1)	AMP AMP/SAM CHL COL FOS MI NAL TI TMP/SMX
	O141:HNM-A-ST10	11-24	1	CTX-M-14 (1)	Stx2e (1)	AMP AMP/SAM ATM CHL COL CTX FEP GEN TI TMP/SMX TOB
	O139:H1-E-ST1	2-54	1	–	Stx2e, F18ab, HlyA (1)	AMP COL MI TI TMP/SMX

*^a^Isolates that did not react with any O antisera were classified as non-typeable (ONT) and non-motile isolates were designated as HNM. PG, phylogroup; ST, sequence type. ^b^CH, clonotype (fumC-fimH alleles); 0 = fimH negative by PCR. ^c^AMP, ampicillin; AMP/SAM, ampicillin-sulbactam; ATM, aztreonam; CAZ, ceftazidime; CHL, chloramphenicol; CIP, ciprofloxacin; COL, colistin; CTX, cefotaxime; FEP, cefepime; FOS, fosfomycin; GEN, gentamicin; LEV, levofloxacin, MI, minocycline; NAL, nalidixic acid; TI, ticarcillin; TMP/SMX, trimethoprim-sulfamethoxazole; TOB, tobramycin. ^∗^Isolates with a MIC value for FOS = 64 (according to EUCAST, the cutoff point is 32 mg/L and higher values are considered resistant, while for CLSI it is 64).*

The antimicrobial susceptibility testing of the 65 *mcr-1* isolates confirmed that all were colistin resistant (MIC of ≥ 4 mg/L). High rates of resistance were also found against ampicillin (75.4%), ticarcillin (73.8%), trimethoprim-sulfamethoxazole (72.3%), ampicillin-sulbactam (64.6%), nalidixic acid (60.0%), and chloramphenicol (58.5%) (Table 4). It is important to note that three isolates were fosfomycin-resistant and, in addition, other eight isolates showed MIC values of 64 mg/L, which is considered as resistant according to EUCAST criteria (MIC breakpoint of 32 mg/L) (EUCAST, 2017). Furthermore, most of the *mcr-1* isolates (61 of 65) met the definition of MDR (Magiorakos et al., 2012) being resistant to at least one agent of ≥3 different antimicrobial categories (Tables 2, 3).

**Table 3:** Molecular characterization of the 24 aEPEC *mcr-1*-positive isolates.

Serotype^a^-PG-ST	CH^b^	No. isolates	Intimin type^c^ (No. isolates)	Resistance profile^d^ (isolates with *mcr* co-occurrence and type)
O2:H40-A-ST10	11-24	4	*eae*-γ2 (3)	AMP AMP/SAM COL TI
				AMP AMP/SAM CHL COL MI TI TMP/SMX
				AMP AMP/SAM CHL COL GEN TI TMP/SMX TOB
			*eae*-Θ2 (1)	CHL COL GEN TMP/SMX TOB
O26:H11-A-ST48	11-54	3	*eae*-𝜀1 (3)	AMP AMP/SAM CHL COL TI
				CHL COL GEN NAL TMP/SMX TOB **(*mcr-1*/*mcr-5*)**
				AMP AMP/SAM COL NAL TI TMP/SMX
O157:H2-A-ST301	27-54	2	*eae*-ξ (2) ^∗^	AMP AMP/SAM CHL COL MI NAL TI TMP/SMX
				AMP AMP/SAM CHL CIP COL GEN MI NAL TI TOB
O45:H2-A-ST301	27-54	1	*eae*-ξ (1)	AMP AMP/SAM COL TI
O157:H2-A-STNew1	27-54	1	*eae*-ξ (1) ^∗^	CHL COL GEN MI NAL TMP/SMX TOB
O26:H11-A-ST7367	685-54	1	*eae*-𝜀1 (1)	COL MI
O103:H2-B1-ST20	4-25	1	*eae*-β1 (1)	AMP AMP/SAM COL TI TMP/SMX
O111:H9-B1-ST29	4-24	2	*eae*-β1 (2)	AMP AMP/SAM CHL COL ^∗^FOS NAL TI TMP/SMX
				CHL COL ^∗^FOS NAL TMP/SMX
O118:H9-B1-ST29	4-24	1	*eae*-β1 (1)	COL NAL TMP/SMX
O123:H11-B1-ST29	4-24	3	*eae*-β1 (3)	AMP CHL COL NAL TI TMP/SMX
				COL MI
				CHL COL NAL TMP/SMX
O177:H11-B1-ST29	4-440	1	*eae*-β1 (1)	AMP AMP/SAM CHL CIP COL GEN LEV MI NAL TI TMP/SMX TOB
O26:H11-B1-ST29	4-440	1	*eae*-β1 (1)	AMP AMP/SAM CHL COL ^∗^FOS TI **(*mcr-1*/*mcr-5*)**
O51:H9-B1-ST29	4-24	1	*eae*-β1 (1)	AMP AMP/SAM CHL COL TI TMP/SMX
O145:H28-E-ST1034	23-331	1	*eae*-γ1 (1)	AMP AMP/SAM CHL COL GEN TI TMP/SMX TOB
O45:H9-E-ST302	84-305	1	*eae*-β1 (1)	AMP AMP/SAM CHL COL GEN MI NAL TI TMP/SMX TOB

*^a^Non-motile isolates were designated as HNM. PG, phylogroup; ST, sequence type. ^b^CH, clonotype (fumC-fimH alleles). ^c∗^HlyA positive isolates. ^d^AMP, ampicillin; AMP/SAM, ampicillin-sulbactam; CHL, chloramphenicol; CIP, ciprofloxacin; COL, colistin; FOS, fosfomycin; GEN, gentamicin; LEV, levofloxacin, MI, minocycline; NAL, nalidixic acid; TI, ticarcillin; TMP/SMX, trimethoprim-sulfamethoxazole; TOB, tobramycin. ^∗^Isolates with a MIC value for FOS = 64 (according to EUCAST, the cut-off point is 32 mg/L and higher values are considered resistant, while for CLSI it is 64).*

**Table 4:** Prevalence of antimicrobial resistances among the 65 *mcr-1* diarrheagenic *E. coli*.

Antimicrobial agent	No. of resistant isolates (%)^a^
Colistin	65 (100)
Ampicillin	49 (75.4)
Ticarcillin	48 (73.8)
Ampicillin-sulbactam	42 (64.6)
Aztreonam	5 (7.7)
Ceftazidime	1 (1.5)
Cefepime	6 (9.2)
Cefotaxime	7 (10.8)
Gentamicin	31 (47.7)
Tobramycin	31 (47.7)
Minocycline	27 (41.5)
Fosfomycin	3 (4.6)^b^
Chloramphenicol	38 (58.5)
Trimethoprim-sulfamethoxazole	47 (72.3)
Nalidixic acid	39 (60.0)
Ciprofloxacin	8 (12.3)
Levofloxacin	7 (10.8)

*^a^Isolates showing intermediate resistance were considered as resistant. None of the 65 mcr-1-positive E. coli isolates showed resistance to piperacillin-tazobactam, imipenem, meropenem, amikacin, or tigecycline. ^b^Additionally, eight isolates showed a MIC value = 64. According to EUCAST, the cut-off point is 32 mg/L and higher values are considered resistant, while for CLSI it is 64.*

Extended-spectrum beta-lactamase genes were detected in six ETEC and one STEC of the 65 *mcr-1* isolates which were typed by sequencing as CTX-M-14 and SHV-12 (six and one isolates, respectively). The PCR screening of other *mcr* variants among the 65 *mcr-1* positive isolates determined that three ETEC isolates carried both *mcr-1*/*mcr-4* genes and other four isolates (two ETEC and two aEPEC) were simultaneous carriers of *mcr-1*/*mcr-5* (Tables 2, 3).

### Phylogroups, STs, Clonotypes, and Virulence-Gene Typing

**FIGURE 1:**
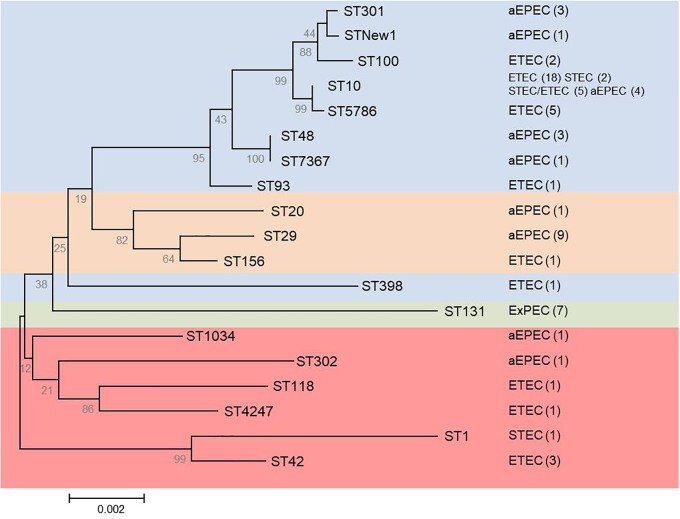
Phylogenetic tree based on concatenated sequences of the seven housekeeping genes from the MLST Achtman scheme by the neighbor-joining method using MEGA6. The analysis includes the 19 STs determined for the *mcr-1* (65 diarrheagenic and seven ST131-B2) positive isolates. Numbers on the tree indicate bootstrap values calculated for 1,000 replicates. Colors indicate: blue (phylogroup A), orange (phylogroup B1), green (phylogroup B2), and red (phylogroup E). Pathotype and number of isolates (in parentheses) are shown on the right.

The 65 *mcr-1* diarrheagenic isolates belonged to phylogroups A (46 isolates), B1 (11 isolates), and E (eight isolates), with 18 different STs including a new one (Figure 1 and Supplementary Table S5). However, more than 50% of these isolates (23 ETEC, eight aEPEC, five STEC/ETEC, and two STEC) were A-ST10 Cplx. The new ST showed by an aEPEC isolate was a single locus variant (SLV) of ST301 and 2 SLV in relation to ST165, so they would be included in the same ST165 Cplx.

Clonotyping identified 19 *fumC*-*fimH* allele combinations (CH), being CH11-24 the most prevalent (29 isolates, 44.6%) which was here associated to STs 10 and 5,796 of the ST10 Cplx, and to different pathotypes (19 ETEC, five STEC/ETEC, four aEPEC, and one STEC). Besides, three of the 65 *mcr-1* isolates were *fimH* negative (Supplementary Table S5).

The F18 and K88 colonizing factors detected in 18 and 12 of the 65 *mcr-1* isolates, respectively, were typed by PCR sequencing. Specifically, the variant K88ac was identified by PCR in the 12 positive isolates, while F18ac and F18ab were determined by sequencing in 17 and one isolate, respectively (Table 2, Supplementary Table S6, and Supplementary Figure S1). The intimin type of the 24 *eae*-positive isolates was also established by sequencing: *eae*-β1 in 11 isolates, *eae*-ξ in four isolates, *eae*-𝜀1 in four isolates, *eae*-γ1 in one isolate, *eae*-γ2 in three isolates, and *eae*-𝜃2 in one isolate (Table 3). All 65 *mcr-1* diarrheagenic isolates were additionally analyzed for the HlyA encoding gene which was detected in 23 ETEC, five STEC/ETEC, two STEC, and three aEPEC (Tables 2, 3).

### Characterization of ST131 Isolates and Detection of *mcr-1* Gene

**FIGURE 2:**
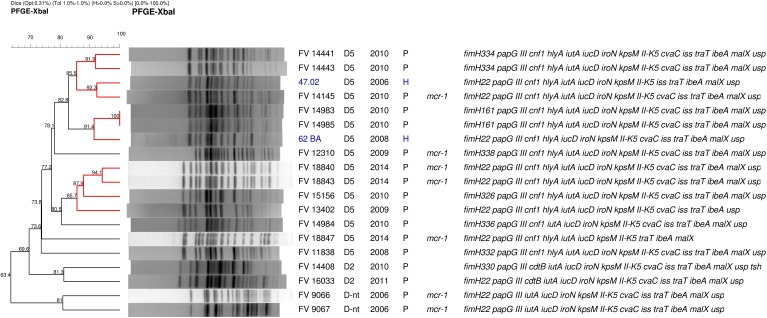
PFGE macrorrestriction profile of 17 pig *E. coli* isolates belonging to the clonal group O25:H4-B2-ST131 (one ST131 isolate resulted autodigested) compared with two human clinical isolates of the LREC collection (showed in blue): association between isolation code, virotype, year, and origin of isolation (P, pig; H, human), *mcr-1* presence, and virulence profile is indicated on the right. Highlighted in red clusters of similarity >85%.

Eighteen isolates recovered by means of the *rfb*O25 screening were confirmed by serotyping, phylogroup and MLST as O25b:H4-B2-ST131. According to their virulence profile, the 18 ST131 isolates belonged to the virotype D, being the majority D5 (13 isolates), and the remaining showed virotypes D2 (two isolates) and D-non typeable (D-nt, three isolates). Importantly, all exhibited the ExPEC and UPEC status. Clonotyping showed eight different clonotypes: CH40-22 (nine isolates), CH40-161 (two), CH40-326 (one), CH40-330 (one), CH40-332 (one), CH40-336 (one), CH40-338 (one), and CH40-374 (two); being five of them (*fimH161*, *fimH326*, *fimH330*, *fimH332*, and *fimH338*) single locus variants of *fimH22* and two of them (*fimH336* and *fimH374*) two loci variants of *fimH22* (Table 5, Supplementary Tables S7, S8, and Supplementary Figures S2, S3). The genetic relatedness and diversity of the ST131 isolates were additionally analyzed by *XbaI* macrorestriction followed by PFGE and compared with human clinical ST131 of our collection (Figure 2). Globally, the pig macrorestriction profiles showed high heterogeneity in the dendrogram where grouped in relation to their virotype with three small cluster identified among D5 clinical isolates (≥85% similarity). Importantly, we detected two human ST131 D5 clinical isolates of high identity (≥85% similarity) with two pig clusters, respectively.

**Table 5:** Phenotypic and genotypic characterization of the 18 O25b:H4-B2-ST131 isolates recovered from the 464 fecal samples of pigs with diarrhea.

CH^a^	Virulence profile^b^	Virotype^c^	No. of isolates/resistance profile^d^	
CH40-22	*fimH22 papEF pap G III iutA iucD iroN kpsM II-K5 cvaC iss traT ibeA malX usp*	D-nt	**1^∗^** **1^∗^**	**AMP AMP/SAM CHL GEN NAL TI TOB TMP/SMXAMP AMP/SAM CHL COL GEN NAL TI TOB TMP/SMX**
	*fimH22 papEF pap G III cnf1 hlyA iutA iucD iroN kpsM II-K5 cvaC iss traT ibeA usp*	D5	1	AMP AMP/SAM NAL TI
	*fimH22 papEF papG III cnf1 hlyA iutA iucD iroN kpsM II-K5 cvaC iss traT ibeA malX usp*	D5	**1^∗^**	**AMP AMP/SAM COL NAL TI TMP/SMX**
	*fimH22 papEF pap G III cdtB iutA iucD iroN kpsM II-K5 cvaC iss traT ibeA malX usp*	D2	1	AMP AMP/SAM MI TI
	*fimH22 papEF pap G III cnf1 hlyA iutA iucD iroN kpsM II-K5 cvaC iss traT ibeA malX usp*	D5	**2^∗^** **1^∗^**	**AMP AMP/SAM CHL COL GEN MI NAL TI TOB TMP/SMXAMP AMP/SAM CIP CHL COL GEN LEV MI NAL TI TOB TMP/SMX**
	*fimH22 papEF pap G III cnf1 hlyA iutA iucD kpsM II-K5 traT ibeA malX*	D5		
	*fimH22 papEF pap G III sfa/focDE cdtB cnf1 hlyA iroN kpsM II-K5 ibeA malX usp*	D-nt	1	-
CH40-161	*fimH161 papEF papG III cnf1 hlyA iutA iucD iroN kpsM II-K5 cvaC iss traT ibeA malX usp*	D5	1	AMP AMP/SAM NAL TI


			1	AMP AMP/SAM TI
CH40-326	*fimH326 papEF pap G III cnf1 hlyA iutA iucD iroN kpsM II-K5 cvaC iss traT ibeA malX usp*	D5	1	AMP AMP/SAM NAL TI
CH40-330	*fimH330 papEF pap G III cdtB iutA iucD iroN kpsM II-K5 cvaC iss traT ibeA malX usp tsh*	D2	1	–
CH40-332	*fimH332 papEF pap G III cnf1 hlyA iutA iucD iroN kpsM II-K5 cvaC iss traT ibeA malX usp*	D5	1	AMP AMP/SAM GEN NAL TI TOB TMP/SMX
CH40-374	*fimH374 papEF pap G III cnf1 hlyA iutA iucD iroN kpsM II-K5 cvaC iss traT ibeA malX usp*	D5	2	AMP AMP/SAM TI
CH40-336	*fimH336 papEF papG III cnf1 iutA iucD iroN kpsM II-K5 cvaC iss traT ibeA malX usp*	D5	1	AMP AMP/SAM CHL NAL TI
CH40-338	*fimH338 papEF pap G III cnf1 hlyA iutA iucD iroN kpsM II-K5 cvaC iss traT ibeA malX usp*	D5	**1^∗^**	**AMP AMP/SAM COL GEN MI NAL TI TOB TMP/SMX**

*^a^CH, clonotype (fumC-fimH alleles). ^b^All isolates exhibited the ExPEC and UPEC status. ^c^nt, not typable. ^d^Resistance profiles of mcr-1 carriers (indicated with asterisk ^∗^) are highlighted in bold. AMP, ampicillin; AMP/SAM, ampicillin-sulbactam; CHL, chloramphenicol; CIP, ciprofloxacin; COL, colistin; FOS, fosfomycin; GEN, gentamicin; LEV, levofloxacin, MI, minocycline; NAL, nalidixic acid; TI, ticarcillin; TMP/SMX, trimethoprim-sulfamethoxazole; TOB, tobramycin;-, sensible to all antibiotics tested.*

Seven CH40-22 isolates out of the 18 ST131 were *mcr-1* carriers. Their antimicrobial susceptibility determined by MIC confirmed that all but one were colistin resistant. Furthermore, the seven showed MDR (Table 5).

## Discussion

Pig diarrhea caused by *E. coli* is a worldwide economically important disease for the swine industry. While ETEC neonatal diarrhea can be effectively controlled by vaccination of pregnant sows, passive protection is quickly lost after weaning (Melkebeek et al., 2013; Matías et al., 2017). Currently, few vaccines are commercially available for PWD, and few provide protection against different *E. coli* pathotypes (Nadeau et al., 2017; Won and John Hwa, 2017). In the present study, we performed a wide epidemiological study on a collection of *E. coli* isolates recovered from 464 faecal samples of enteric colibacillosis in pig production farms of Spain over a 10-year period to know the *E. coli* traits (pathotypes) presently implicated in swine diarrheas, and how the use of antibiotics (including colistin) could have affected the selection and emergence of resistant strains. Most samples (73%) were of diarrheagenic cases occurred during the post-wean period which showed to be significantly associated with ETEC (67%) followed by aEPEC (21.7%). By contrast, aEPEC was more prevalent (60.3%) among diarrheas of suckling piglets, followed by ETEC (38.8%). STEC/ETEC or STEC were recovered in 11.3 and 0.9% of PW and neonatal diarrhea, respectively. It is noteworthy that the F4 colonization factor was identified among neonatal ETEC without significant differences compared to PWD ETEC (40.5 versus 27.7%) while, as expected, F18 was only present among PWD isolates.

Complete data on the prevalence, serotypes, and pathotypes are not frequently available, which makes comparisons difficult. However, previous analysis had already suggested differences between countries (Frydendahl, 2002; Blanco et al., 2006; Vu Khac et al., 2006). A recent study across Europe regarding ETEC and STEC PWD pathotypes describes a higher prevalence of F4 compared to F18 isolates in Belgium and The Netherlands, France, and Italy (Luppi et al., 2016). Although pairwise comparisons for each country gave no significant differences, the epidemiologic situation of PWD is clearly different from that found in our study for Spain (23.9% F4 isolates versus 51.5% F18 of ETEC, STEC, and STEC/ETEC isolates; *P* < 0.001). The reported association of F18 *E. coli* isolates with PWD in other countries varied widely, from 1.7% in Australia (Smith et al., 2010), 35% in Slovakia (Vu Khac et al., 2006), 39.3% in Denmark (Frydendahl, 2002), 53% in United States (Post et al., 2000), to 62% in Poland (Osek et al., 1999). High F18 prevalence (62.9% of 648 isolates) was as well reported in a study performed in Japan where, in addition, most isolates carried *stx_2e_* gene (60.1% of 648 isolates) (Kusumoto et al., 2016) which describes a very different pathogenic profile in relation to other geographic areas. Thus, we found 10% of *stx*_2e_-positive isolates among the Spanish collection of 481 *E. coli*, similar to the numbers reported in the European study of Luppi et al. (2016). Like the Japanese study, most of our *stx*_2e_ isolates (73.9%) were positive for F18.

The majority of ETEC associated with diarrhea in pigs appear to belong to a limited number of serogroups, being O8, O138, O139, O141, O147, O149, and O157 the most commonly reported worldwide. Similarly, STEC causing disease primarily belong to O138, O139, and O141 (Nagy and Fekete, 1999; Frydendahl, 2002; Fairbrother et al., 2005). The majority of ETEC and STEC isolates analyzed in this work belonged to the most common serogroups O8, O138, O139, O141, O147, O149, and O157. In addition, O15, O35, O45, O101, and O108 serogroups, less frequently reported, were also present within the ETEC isolates ranging between 15.5% for O108 and 1.8% for O35; likewise, STEC single isolates presented the infrequent O2, O22, O36, O142, and O158 serogroups. Apart from the 50 serogroups determined within 337 isolates, it is of note that other 144 (23.7%) isolates of different pathotypes did not react with any of the O1 to O185 antisera and remained as non-typeable (ONT).

We found in our study a high prevalence of aEPEC (156 out of 481; 32.4%) as presumptive agents of the clinical condition, and significantly associated with neonatal samples. For atypical EPEC, both animals and humans can be reservoirs and are known as pathogenic for children and young animals (Trabulsi et al., 2002; Watson et al., 2017). aEPEC have also been implicated in PWD in pigs but their pathogenicity still remains unclear (Malik et al., 2017). According to these and other authors, there exist differences of serotypes and intimin types among porcine *eae*-positive isolates in relation to the pathogenic potential, such as sero/intimin type O123:H11/*eae-β1* or O45/*eae-β1* (both determined in our collection) with AE activity on ileal villi and frequently occurring in diarrheagenic pigs (Zhu et al., 1994; Malik et al., 2017). In previous studies on swine diarrhea in Slovakia, we had found a much lower aEPEC involvement (3.2 and 0.9% of neonatal and PWD isolates, respectively) (Vu Khac et al., 2006, Vu Khac et al., 2007).

Since the discovery of the *mcr-1* gene in late 2015, its detection has been reported globally, but its detection rate has been variable depending on the geographic region, source, and method of identification. Overuse of colistin in food animals is believed to have trigged the emergence and spread of *mcr-1*, consistently with the higher figures found in poultry and pig industry (Sun et al., 2018). Here, the characterization of 65 representative *mcr-1* isolates, including 33 ETEC, 24 aEPEC, five STEC/ETEC, and three STEC showed that all were phenotypically resistant to colistin, and most (61 of 65) met the MDR definition of Magiorakos et al. (2012). MDR in pig industry has been previously reported in different studies, and associated with the widely use of aminoglycosides and beta-lactams in veterinary medicine (Rhouma et al., 2016). The 37 *mcr-1* positive isolates recovered from PWD in Italy also showed genetically diverse pathotypes and all but one were resistant to ≥3 different antimicrobial families (Curcio et al., 2017). Li et al. (2017) found a higher prevalence of *mcr-1* positie isolates among pathogenic *E. coli* from diseased pigs than from healthy pigs (45.1 versus 15.7%, *P* = 0.000); besides, resistance profiles of *mcr-1* positive *E. coli* were more extensive than those of *mcr-1* negative isolates. In our study, it is also of concern the finding of 11 out of the 65 *mcr-1* isolates with MIC >32 mg/L for fosfomycin. Fosfomycin resistance is uncommon and primarily associated with specific chromosomal mutations, however plasmid-mediated resistance in livestock has been detected in Asian countries (Chan et al., 2014), and quite recently in France (Lupo et al., 2018). This antibiotic is considered an “old” broad-spectrum antibiotic such as colistin, reconsidered as a good candidate for treating infections caused by MDR microorganisms (Dijkmans et al., 2017).

Six ETEC and one STEC *mcr-1* isolates were also carriers of ESBL genes. In addition, other seven *mcr-1* isolates showed to harbor *mcr-4* (three ETEC) and *mcr-5* (two ETEC and two aEPEC) genes. We have not investigated if the double carriage of our isolates was in the same or different plasmids. Previous studies have reported the co-existence on the same plasmid of other resistance genes, such as *bla_CTX-M_*, *bla_CMY_*, *bla_TEM_*, *fosA*, *qnrS*, *floR*, and *oqxAB*, in various combinations, alongside *mcr-1* (Li R. et al., 2017). At the same time, new *mcr* genes have been successively discovered in plasmids of different origins, as well as co-transfer of variants in promiscuous plasmids (Sun et al., 2018). Since colistin is commonly thought as the last line antibiotic for the treatment of infections caused by MDR and extensively drug-resistant Gram-negative pathogens, such as carbapenem-resistant *Enterobacteriaceae*, it must be beared in mind that its use could lead to co-selection of colistin with other antibiotic-resistant isolates.

Of the 41 ETEC, STEC and STEC/ETEC *mcr-1* isolates (Table 2), 18 were carriers of F18. Three F18 subtypes have been reported so far (F18ab, F18ac, and F18New) differentiated by five amino acids at specific positions (31, 57, 59, 83, and 122) with strong association between subtypes and pathotypes (Bosworth et al., 1998; DebRoy et al., 2009; Barth et al., 2011; Byun et al., 2013). Here, the F18 positive isolates subtyped as F18ac corresponded with the ETEC and STEC/ETEC pathotypes (15 and two isolates, respectively) while F18ab was detected in a single STEC isolate. Furthermore, the predicted amino acid sequences of the *fedA* genes identified in this study together with 13 sequences from Barth et al. (2011) were compared and their relatedness analyzed, demonstrating its polymorphic variability (Bosworth et al., 1998).Thus, 10 different amino acid sequences were found among the F18ac subtype of our collection, one of them (detected in four isolates) showed 100% similarity with the GQ325624 sequence identified by Barth et al. (2011) in Germany (Supplementary Figure S1). Interestingly, the deduced amino acid sequence of one isolate (FV18854) presented a novel glycine residue in position 59; however, it was classified as F18ac since the other four amino acids positions are equal to those described for this subtype (Supplementary Table S6) and clustered together with F18ac group in the phylogenetic tree (Supplementary Figure S1).

We found high diversity in the phylogenetic analysis of the 65 *mcr-1* diarrheagenic isolates; however, more than 50% (38 out of 65) belonged to A-ST10 Cplx and from those, 29 isolates of different pathotypes showed CH11-24. The ST10 Cplx of *E. coli*, widely disseminated among animal and human intestinal samples both as a commensal or as a pathogen, is commonly encountered as antimicrobial susceptible but also linked with MDR and ESBL, and recognized as an emerging food-borne ExPEC lineage (Manges and Johnson, 2012). A recent study on genotyping of 68 *mcr-1-*like-positive *E. coli* from food animals at slaughter in Europe between 2002 and 2014, found a high genetic diversity among the isolates with 38 different STs, but being ST10 Cplx and, specifically the ST10, the most prevalent (El Garch et al., 2017, 2018). This finding is also consistent with the observation of Matamoros et al. (2017) who stated that within the overall diversity in the *E. coli* population, two lineages (ST10 and ST155) might function as reservoirs of the *mcr-1* gene, the largest of which was linked to ST10 (Matamoros et al., 2017). In our particular case, it is also important to consider that ST10 Cplx is one of the main *E. coli* clonal complexes associated with porcine ETEC. In fact, Shepard et al. (2012) found that the majority of the porcine ETEC isolates belonged to three clonal complexes: 10, 23, and 165, and pointed out the association showed by ST10 Cplx and ST23 Cplx with certain resistance-associated elements, such as AmpC-type beta-lactamases, NDM-type carbapenemases, and other ESBLs. Other STs established in the present study such as ST1, ST29, ST42, ST100, or ST4247 have been previously associated to enteric pathogenic *E. coli* in pigs (Abraham et al., 2014; Kusumoto et al., 2016).

Interestingly, nine of the 24 aEPEC *mcr-1* characterized in this work belonged to B1-ST29 and carried *eae-β1* gene. They showed six serogroups, including O26, with two H-antigen combinations (H9 or H11). Additionally, two aEPEC belonged to the clonal groups O103:H2-B1-ST20 (*eae-β1*) and O145:H28-E-ST1034 of the ST32 Cplx (*eae-γ1*), respectively. Enterohemorrhagic *E. coli* (EHEC) is the causative agent of bloody diarrhea, the hemolytic-uremic syndrome (HUS), and thrombotic thrombocytopenic purpura (TTP). Besides the intimin (Eae) which confers the ability to cause AE lesions, EHEC harbors bacteriophage-encoded *stx* genes. Currently, the vast majority of EHEC infections are caused by isolates belonging to five O serogroups, namely O157, O26, O103, O111, and O145 (Mellmann et al., 2008). Eichhorn et al. (2015) analyzed by MLST the phylogenetic relationships in a collection of 250 isolates belonging to serogroups O26, O103, O111, and O145 defined as EHEC, obtained from different sources of isolation. As a result, the majority of the O26 and O111 EHEC isolates clustered into the ST29 Cplx. O103 isolates clustered mainly in ST20 Cplx, and most isolates of O145 were found within ST32 Cplx. In addition to EHEC, the ST29 Cplx cluster also included aEPEC isolates. According to these authors, the finding that aEPEC and EHEC isolates of non-O157 serogroups share the same phylogeny suggests an ongoing microevolutionary scenario in which the phage-encoded *stx* is transferred between aEPEC and EHEC. The concept of interconversion between STEC and aEPEC had been previously suggested by Bielaszewska et al. (2007) for O26 isolates by the loss, as well as by the gain, of the *stx*-encoding prophage through the lysogenic conversion. Applying the concept of bidirectional conversion, it could be hypothesized that the EPEC strains could function as pre-STEC strains that integrate the *stx* prophage into their genomes. However, this hypothesis together with the potential human pathogenicity of EPEC isolates from sources such as swine requires extensive research.

A highly important finding of the present study was the recovery of 18 ST131 isolates among the 464 fecal samples of diarrheanic pigs. As far as we know, there are only two reports of pig origin for this clonal group: the first obtained from pork meat in Denmark in 2003 (Trobos et al., 2009), and the second was a CTX-M-1 ST131 recovered from a gastrointestinal tract infection in a pig among 1,378 isolates analyzed in the frame of an ESBL monitoring program performed in Germany during the years 2006–2007 (Schink et al., 2013). None of the 18 ST131 detected in this work were carriers of ESBL genes but instead, most were MDR, harbored 12–15 virulence-gene traits and, surprisingly, seven isolates were *mcr-1* carriers. Very few *mcr-1* isolates have been reported belonging to the pandemic clone ST131, responsible for the high incidence of ExPEC infections as well as the worldwide dissemination of multidrug resistance (Nicolas-Chanoine et al., 2008, 2014). So far, ST131 carrying *mcr-1* was first described in an isolate from chicken meat (Hasman et al., 2015), then in poultry (Ewers et al., 2016) and in a few human clinical isolates (Kuo et al., 2016; Sonnevend et al., 2016; Ortiz de la Tabla et al., 2017). To the best of our knowledge, this would be the first report of ST131 *mcr-1* isolation in pigs. The antimicrobial susceptibility of the seven *mcr-1* ST131 swine isolates of this work determined by MIC, confirmed that all but one isolate were colistin resistant. The presence of the *mcr-1* gene in susceptible isolates has been described (Fernandes et al., 2016; El Garch et al., 2017). Here, we investigated the *mcr-1* gene from the susceptible isolate with primers listed in Supplementary Table S3, and failed when trying to obtain the entire gene sequence, suggesting the gene is truncated or has been modified by agents such as insertion sequences like those previously reported (Terveer et al., 2017; Zhou et al., 2018); however, further work is necessary to determine the cause of loss of function. Besides, the seven *mcr-1* ST131 swine isolates showed resistance against 6–12 antibiotics, including a ciprofloxacin-resistance determined in 1 isolate (Table 5). In a nationwide study performed in Spain during 2005–2012 in five hospitals of different regions, the ST131 accounted for 490 (16%) of the 2,995 isolates obtained from clinical human samples (Dahbi et al., 2014). The majority (78%) of the ST131 isolates belonged to the recently emerged fluoroquinolone-resistant ST131-*H30*R subclone and 61.6% to the *H30*-Rx additionally carrier of CTX-M15, while ST131-*H22* subclone was the second most prevalent of the human collection. Dahbi et al. (2014) found in their work different patterns of associated antimicrobial resistances in relation to the *fimH* allele (*fimH*30 or *fimH*22). Ciprofloxacin, trimethoprim-sulfamethoxazole, gentamicin, and tobramycin resistances were significantly associated with *fimH30* isolates in comparison with *fimH*22 isolates. In fact, they only detected one and eight isolates resistant to ciprofloxacin and trimethoprim-sulfamethoxazole, respectively, among 20 ST131-*H22*. Dahbi et al. (2014) also found association of the *fimH* alleles with the virulence profile, being all 20 *fimH*22 isolates of virotype D, subtypes D1, D2, D4, and D5. In the present work, we found high genetic variability within the eight *fimH* alleles of 18 ST131 isolates; however, *fimH22* was the most prevalent (nine isolates) and the other seven alleles were *fimH22* variants whose nucleotide and aminoacidic differences were compared with those reported by Dahbi et al. (2014) (Supplementary Tables S7, S8 and Supplementary Figures S2, S3). All our swine isolates conformed the D virotype and showed D5 (13 isolates) and D2 (two isolates) also described within the human collection. Worryingly, the seven swine ST131 carriers of *mcr-1* also showed a higher number of resistances, including to trimethoprim-sulfamethoxazole (seven isolates), tobramycin (six isolates), gentamicin (six isolates), and ciprofloxacin (one isolate), more associated to the *fimH30* subclone according to Dahbi et al. (2014). It is also remarkable that the 18 ST131 swine isolates exhibited virulence traits that satisfied the ExPEC and UPEC status according to Johnson et al. (2003) and Spurbeck et al. (2012) definitions. In the PFGE macrorestriction, comparison of our swine isolates with those of clinical human origin, two pig isolates clustered with two human ST131 D5 showing high similarities (≥85%).

## Conclusion

In conclusion, the comprehensive characterization of this wide collection of *E. coli* isolates recovered from enteric swine colibacillosis in Spain provides valuable epidemiological information about the current pathotypes involved in this economically important pathology, as well as alerts about the worrisome presence of MDR clones such as ST131. Acquisition of *mcr-1* by this specific clone means an increased risk due to its special feature of congregating virulence and resistance traits, together with its spread capability. Here, we give evidences of the potential human pathogenicity of ST131 D5 isolates of swine origin due to their genetic identity.

## Author Contributions

AM and JB conceived and designed the experiments. IG-M, VG, DD-J, SF-S, and MA performed the experiments. AM, JB, VG, IG-M, JB, and MB analyzed and interpreted the data. AM, VG, JB, and IG-M drafted the manuscript. AM, JB, VG, IG-M, JB, MB, MA, DD-J, and SF-S provided critical input and approved the final manuscript.

## Conflict of Interest Statement

The authors declare that the research was conducted in the absence of any commercial or financial relationships that could be construed as a potential conflict of interest.

## Footnotes

1 http://enterobase.warwick.ac.uk/species/ecoli/allele_st_search

2 https://bitbucket.org/genomicepidemiology/fimtyper_db/downloads/

3 http://www.pulsenetinternational.org/
