# Supplementary material for: Swine Enteric Colibacillosis in Spain: Pathogenic Potential of mcr-1 ST10 and ST131 E. coli Isolates
Source: Front Microbiol. 2018 Nov 5;9:2659. doi: 10.3389/fmicb.2018.02659 (PMC6230658; doi:10.3389/fmicb.2018.02659)
Supplement: Supplementary file 1 [file Data_Sheet_1.PDF]

Supplementary Material

Swine enteric colibacillosis in Spain: pathogenic potential of *mcr-1* ST10 and ST131 *E. coli* isolates

Isidro García-Meniño, Vanesa García, Azucena Mora[*], Dafne Díaz-Jiménez, Saskia C. Flament-Simon, María Pilar Alonso, Jesús E. Blanco, Miguel Blanco, Jorge Blanco

*** Correspondence:** azucena.mora@usc.es

Table S1. Targets and primers associated with diarreagenic and extraintestinal pathotypes of *E. coli*

Pathotype	Target	Primers	Nucleotide sequence (5´- 3´)	Size (bp)	Reference
STEC	*stx1*	VT1-F	TCGCTGAATGTCATTCGCTCTGC	539	Mora *et al.*, 2011
		VT1-R	TCAGCAGTCATTACATAAGAAC		
STEC	*stx2*	VT2-F1	TTTCTTCGGTATCCTATTCCC	358	Mora *et al.*, 2011
		VT2-F2	TGTCTTCAGCATCTTATGCAG		
		VT2-R	CTGCTGTCCGTTGTCATGGAA		
STEC	*stx2e*	Stx2e-F1	CGGAGTATCGGGGAGAGGC	411	Scheutz *et al.*, 2012
		Stx2e-R2	CTTCCTGACACCTTCACAGTAAAGGT		
STEC EPEC	*eae*	EAE-V3F	CATTGATCAGGATTTTTCTGGT	510	Mora *et al.*, 2011
		EAE-MBR	TCCAGAATAATATTGTTATTACG		
STEC EPEC	*eae*	[a]EAE-R11	TCTTCGGAGGGTTTTTTATT	1125	Alonso *et al.*, 2017
		[a]EAE-FBN	CAGGTCGTCGTGTCTGCTAAAAC		
STEC EPEC	*eae*	[a]EAE-R12	CCAGACGAATATATACATATTC	1181	Alonso *et al.*, 2017
		[a]EAE-FBN	CAGGTCGTCGTGTCTGCTAAAAC		
tEPEC	*bfpA*	BFP-NF1	ATGGTTTCTAAAATCATGAATAAG	262	Bennett, 2003
		BFP-NR1	ATTATTCCGGAATTGCAGATGTGT		This study
ETEC	*eltA*	LT-A-1	GGCGACAGATTATACCGTGC	696	Schultsz *et al.*, 1994
		LT-A-2	CCGAATTCTGTTATATATGTC		
ETEC	*estA*	STa-A	ATTTTTATTTCTGTATTGTCTTT	176	Penteado *et al.*, 2002
		STa-B	GGATTACAACACAGTTCACAGCAGT		
ETEC	*estB*	STb-1	ATCGCATTTCTTCTTGCATC	172	Blanco *et al.*, 1997
		STb-2	GGGCGCCAAAGCATGCTCC		
ETEC	F18	[b]fedA-1	GTGAAAAGACTAGTGTTTATTTC	511-514	Imberechts *et al.*, 1992
		[b]fedA-2	CTTGTAAGTAACCGCGTAAGC		
ETEC	F4 (K88)	K88-F	GGTGATTTCAATGGTTCGGTC	764	Franklin *et al.*, 1996
		K88-R	ATTGCTACGTTCAGCGGAGCG		
ETEC	F4 (K88ab)	K88-F	GGTGATTTCAATGGTTCGGTC	501	Franklin *et al.*, 1996
		K88-ab	TGCAGCACCCGAAACAGTCGTCGT		
ETEC	F4 (K88ac)	K88-F	GGTGATTTCAATGGTTCGGTC	507	Franklin *et al.*, 1996
		K88-ac	CCCAGCCGACGATTCAGAACCCCT		
ETEC	F4 (K88ad)	K88-F	GGTGATTTCAATGGTTCGGTC	501	Franklin *et al.*, 1996
		K88-ad	TGCAGAATTCTGAACATTCGTCGG		
ETEC	F5 (K99)	K99-A	CCAGCGCCCGGCAGTAATGACTGC	278	Blanco *et al.*, 2006
		K99-B	CCACCATTAGACGGAGCGCGG		
ETEC	F41	F41-A	GGCTATGGAAGACTGGAGAGGG	545	Blanco *et al.*, 2006
		F41-RN	GACTGAGGTCATCCCAATTGTGG		This study
ETEC	F6 (P987)	P987-F	GCGCCCGCTGAAAACAACACCAGC	467	Blanco *et al.*, 2006
		P987-R	GTACCGGCCGTAACTCCACCG		
ExPEC	*fimH*	FimH-f	TGCAGAACGGATAAGCCGTGG	508	Johnson and Stell, 2000
		FimH-r	GCAGTCACCTGCCCTCCGGTA		
ExPEC	*fimAv*MT78	fimA201	TCTGGCTGATACTACACC	266	Marc and Dho-Moulin, 1996
		fimA215	ACTTTAGGATGAGTACTG		

Pathotype	Target	Primers	Nucleotide sequence (5´- 3´)	Size (bp)	Reference
ExPEC	*papC*	Forward	GTGGCAGTATGAGTAATGACCGTTA	205	Johnson *et al.*, 2015
		Reverse	ATATCCTTTCTGCAGGGATGCAATA		
ExPEC	*papAH*	papA-F	ATGGCAGTGGTGTCTTTTGGTG	720	Johnson and Stell, 2000
		papA-R	CGTCCCACCATACGTGCTCTTC		
ExPEC	*papEF*	PapEF-F	GCAACAGCAACGCTGGTTGCATCAT	336	Yamamoto *et al.*, 1995
		PapEF-R	AGAGAGAGCCACTCTTATACGGACA		
ExPEC	*papG I*	pap-I F	TTAGCTGGATGGCACAATG	335	Mora *et al.*, 2013
		pap-I R	TTGTCCATGTATCCCATTCAT		
ExPEC	*papG II*	pap-II F	GGGCATTGCTACGGTAACCTG	545	Mora *et al.*, 2013
		pap-II R	CGCTATTAATAGACAGATCACC		
ExPEC	*papG III*	pap-III F	CGGCAACTTTAAGCTATGTG	720	Mora *et al.*, 2013
		pap-III R	TGTACCATCTCATCGTTGTCTC		
ExPEC	*sfa/focDE*	sfa1	CTCCGGAGAACTGGGTGCATCTTAC	410	Le Bouguenec *et al.*, 1992
		sfa2	CGGAGGAGTAATTACAAACCTGGCA		
ExPEC	*afa/draBC*	afa1	GCTGGGCAGCAAACTGATAACTCTC	750	Le Bouguenec *et al.*, 1992
		afa2	CATCAAGCTGTTTGTTCGTCCGCCG		
ExPEC	*cnf1*	cnf1-f2	CAGGAGGTACTTAGCAGCGT	468	Mora *et al.*, 2013
		cnf1-rc	TAATTTTGGGTTTGTATC		
ExPEC	*cdtB*	cdt-s1	GAAAGTAAATGGAATATAAATGTCCG	466	Tóth *et al.*, 2003
		cdt-as1	AAATCACCAAGAATCATCCAGTTA		
		cdt-s2	GAAAATAAATGGAACACACATGTCCG		
		cdt-as2	AAATCTCCTGCAATCATCCAGTTA		
ExPEC	*sat*	SatF	GCAGCTACCGCAATAGGAGGT	937	Johnson *et al.*, 2003
		SatR	CATTCAGAGTACCGGGGCCTA		
ExPEC	*hlyA*	hly F	AACAAGGATAAGCACTGTTCTGGCT	1177	Yamamoto *et al.*, 1995
		hly R	ACCATATAAGCGGTCATTCCCGTCA		
ExPEC	*iucD*	Aer F	TACCGGATTGTCATATGCAGACCGT	602	Yamamoto *et al.*, 1995
		Aer R	AATATCTTCCTCCAGTCCGGAGAAG		
ExPEC	*iroN*	Ironec-F	AAGTCAAAGCAGGGGTTGCCCG	665	Johnson *et al.*, 2000
		Ironec-R	GACGCCGACATTAAGACGCAG		
ExPEC	*kpsM II*	KpsII f	GCGCATTTGCTGATACTGTTG	272	Johnson and Stell, 2000
		KpsII r	CATCCAGACGATAAGCATGAGCA		
ExPEC	*kpsM II-K2*	KpsII f	GCGCATTTGCTGATACTGTTG	570	Johnson and O'Bryan, 2004
		KpsII-K2r	AGGTAGTTCAGACTCACACCT		
ExPEC	*kpsM II-K5*	K5-f	CAGTATCAGCAATCGTTCTGTA	159	Johnson and Stell, 2000
		KpsII r	CATCCAGACGATAAGCATGAGCA		
ExPEC	*neuC*	neu1	AGGTGAAAAGCCTGGTAGTGTG	676	Moulin-Schouleur *et al.*, 2006
		neu2	GGTGGTACATCCCGGGATGTC		
ExPEC	*kpsM III*	KpsIII f	TCCTCTTGCTACTATTCCCCCT	392	Johnson and Stell, 2000
		KpsIII r	AGGCGTATCCATCCCTCCTAAC		
ExPEC	*cva*C	ColV-CF	CACACACAAACGGGAGCTGTT	680	Johnson and Stell, 2000
		ColV-CR	CTTCCCGCAGCATAGTTCCAT		

Pathotype	Target	Primers	Nucleotide sequence (5´- 3´)	Size (bp)	Reference
ExPEC	*traT*	TraT f	GGTGTGGTGCGATGAGCACAG	290	Johnson and Stell, 2000
		TraT r	CACGGTTCAGCCATCCCTGAG		
ExPEC	*ibeA*	ibe10 f	AGGCAGGTGTGCGCCGCGTAC	170	Johnson and Stell, 2000
		ibe10 r	TGGTGCTCCGGCAAACCATGC		
ExPEC	*malX*	MALX-F	GCATGAGCAGTGCGATACATCGC	828	Mora *et al.*, 2013
		MALX-R	AGGGCTGGGAAGTGGTTTAGCC		
ExPEC	*usp*	usp-F	ACATTCACGGCAAGCCTCAG	440	Bauer *et al.*, 2002
		usp-R	AGCGAGTTCCTGGTGAAAGC		
ExPEC	*iutA*	aer-851F	GGCTGGACATCATGGGAACTGG	301	Johnson *et al.*, 1997
		aer-1152R	CGTCGGGAACGGGTAGAATCG		
ExPEC	*tsh*	tsh03	GGTGGTGCACTGGAGTGG	640	Dozois *et al.*, 2000
		tsh15	AGTCCAGCGTGATAGTGG		
UPEC	*vat*	vat-F	TCAGGACACGTTCAGGCATTCAGT	1100	Spurbeck *et al.*, 2012
		vat-R	GGCCAGAACATTTGCTCCCTTGTT		
UPEC	*fyuA*	fyuA-F	GTAAACAATCTTCCCGCTCGGCAT	850	Spurbeck *et al.*, 2012
		fyuA-R	TGACGATTAACGAACCGGAAGGGA		
UPEC	*yfcV*	yfcV-F	ACATGGAGACCACGTTCACC	292	Spurbeck *et al.*, 2012
		yfcV-R	GTAATCTGGAATGTGGTCAGG		
UPEC	*chuA*	ChuA-F	CTGAAACCATGACCGTTACG	652	Spurbeck *et al.*, 2012
		ChuA-R	TTGTAGTAACGCACTAAACC		

[a] Primers used for the *eae* typing (amplification and sequencing).
[b] Primers used for the detection of F18 and its variants (amplification and sequencing).

Table S2. Primers used for H antigen typing

Target	Primers	Nucleotide sequence (5´- 3´)	Size (bp)	Reference
*fli*C$_{H1}$	H1-F2	TATCCGGTCAGACCCAGTTC	828	This study
	H1-R2	TTGCGGATGTATCACCGTTA		
*fli*C$_{H2}$	H2-F	AACGACGGCGAAACAATTAC	828	Alonso *et al*., 2017
	H2-R	AGAACGCAACGAGTCAACCT		
*fli*C$_{H4}$	H4-F	GCAGCGTATTCGTGAACTGA	713	Mora *et al*., 2011
	H4-R	GCTGGATAATCTGCGCTTTC		
*fli*C$_{H7}$	H7-F	GCGCTGTCGAGTTCTATCGAGC	625	Gannon *et al*., 1997
	H7-R	CAACGGTGACTTTATCGCCATTCC		
*fli*C$_{H8}$	H8-F	TAACAGCGCAAAAGACGATG	393	Mora *et al*., 2012
	H8-R	CCGAGAGTTTTCGCATCAAT		This study
*fli*C$_{H9}$	H28-F	ACGAAATCAAATCCCGTCTG	649	Mora *et al*., 2012
	H9-R	GCGGTATCGTTACCTGCATT		This study
*fli*C$_{H10}$	H10-F	AGCAAGTGGCAGTAGGTGCT	624	Alonso *et al*., 2017
	H10-R	GCTGGATAATCTGCGCTTTC		
*fli*C$_{H11}$	H11-F	ACTGTTAACGTAGATAGC	248	Durso *et al*., 2005
	H11-R	TCAATTTCTGCAGAATATAC		
*fli*C$_{H18}$	H18-F1	TTCTGACCTGGACTCCATCC	827	Mora *et al*., 2018
	H18-R1	CGTTAGCAAACGTTGAAGCA		
*fli*C$_{H21}$	H21-F	GGCGATTGCTAACCGTTTTA	549-556	Mora *et al*., 2012
	H21-R3	CGTAAGTGAACCATCCGCAG		
*fli*C$_{H25}$	H25-F	ATGAAATTGACCGCGTATCC	212	Alonso *et al*., 2017
	H25-R	TTGCGGGATAGATGTGATAGC		
*fli*C$_{H28}$	H28-F	ACGAAATCAAATCCCGTCTG	856	Mora *et al*., 2012
	H28-R	GCCGATTGAAGAGACTCAGC		

Table S3. Primers used for the detection and / or sequencing of TEM, SHV, CTX-M and MCR genes

Target	Primers	Nucleotide sequence (5´- 3´)	Size (bp)	Reference
*bla*CTX-M	CTX-C3	ATGTGCAGCACCAGTAAAGTGATG	542	Mora *et al.*, 2013
	CTX-C4	ACCGCGATATCGTTGGTGGTGCC		
*bla*CTX-M group1	M13U	GGTTAAAAAATCACTGCGTC	863	Saladin *et al.*, 2002
	M13L	TTGGTGACGATTTTAGCCGC		
*bla*CTX-M group9	[a]CTX-M9-F	GTGACAAAGAGAGTGCAACGG	856	Simarro *et al.*, 2000
	[a]CTX-M9-R	ATGATTCTCGCCGCTGAAGCC		
*bla*CTX-M group9	[b]CTX-M9-14-14B-24F	GAATACTGATGTAACACGGA	998	This study
	[b]CTX-M9-R	AGCTGAAGATGTATATCAAG		
*bla*CTX-M group9	[b]CTX-M9-14-14B-24F	GAATACTGATGTAACACGGA	989	This study
	[b]CTX-M14-24-R	CTGCGTTGTCGGGAAGATACG		
*bla*CTX-M group9	[b]CTX-M9-14B-F	CCTATACCCGAGGCGCGACAG	1059	This study
	[b]CTX-M9-R	AGCTGAAGATGTATATCAAG		
*bla*CTX-M group9	[b]CTX-M14-24-F	CTAAATTCTTCGTGAAATAGTG	1049	This study
	[b]CTX-M14-24-R	CTGCGTTGTCGGGAAGATACG		
*bla*SHV	SHV-F2	TTGTCGCTTCTTTACTCGCC	879	Mora *et al.*, 2013
	SHV-R2	CCCGGCGATTTGCTGATTTCGC		
*bla*SHV	[b]SHV-1	GGGTTATTCTTATTTGTCGC	930	Rasheed *et al.*, 1997
	[b]SHV-2	TTAGCGTTGCCAGTGCTC		
*bla*TEM	[a]TEM-1-F	ATGAGTATTCAACATTTCCG	868	Rasheed *et al.*, 1997
	[a]TEM-1-R	CTGACAGTTACCAATGCTTA		
mcr-1	CLR5-F	CGGTCAGTCCGTTTGTTC	309	Liu *et al.*, 2016
	CLR5-R	CTTGGTCGGTCTGTAGGG		
mcr-1	[b]mcrS1-F	GGGATTGCGCAATGATTGC	548	This study
	[b]mcrS1-R	CACCCAAACCAATGATACG		
mcr-1	[b]mcrS1-F2	AAAGACGCGGTACAAGCAAC	1016	This study
	[b]mcrS1-R2	CCCACCGCCCATAATACGAA		
mcr-2	mcr-2 IF	TGTTGCTTGTGCCGATTGGA	567	Xavier *et al.*, 2016
	mcr-2 IR	AGATGGTATTGTTGGTTGCTG		
mcr-3	MCR3-F	TTG GCACTGTATTTTGCATTT	542	Yin *et al.*, 2017
	MCR3-R	TTAACGAAATTGGCTGGAACA		
mcr-4	mcr-4 FW	ATTGGGATAGTCGCCTTTTT	487	Carattoli *et al.*, 2017
	mcr-4 RV	TTACAGCCAGAATCATTATCA		
mcr-5	MCR5_FW	ATGCGGTTGTCTGCATTTATC	1644	Borowiak *et al.*, 2017
	MCR5_RV	TCATTGTGGTTGTCCTTTTCTG		

[a] Primers used for amplification and sequencing.
[b] Primers used for sequencing.

Table S4. Targets and primers to determine phylogroups, clonotypes and sequence types by MLST

Target	Primers	Nucleotide sequence (5´- 3´)	Size (bp)	Reference
**Quadruplex phylotyping method of Clermont *et al.*, 2013**				
chuA	chuA.1b	ATGGTACCGGACGAACCAAC	288	Clermont *et al*., 2013
	chuA.2	TGCCGCCAGTACCAAAGACA		Clermont *et al*., 2000
yjaA	yjaA.1b	CAAACGTGAAGTGTCAGGAG	211	Clermont *et al*., 2013
	yjaA.2b	AATGCGTTCCTCAACCTGTG		
TspE4C2	TspE4C2.1b	CACTATTCGTAAGGTCATCC	152	Clermont *et al*., 2013
	TspE4C2.2b	AGTTTATCGCTGCGGGTCGC		
arpA	AceK.f	AACGCTATTCGCCAGCTTGC	400	Clermont *et al*., 2013
	ArpA1.r	TCTCCCCATACCGTACGCTA		
trpAgpC	trpAgpC.1	AGTTTTATGCCCAGTGCGAG	219	Lescat *et al*., 2013
	trpAgpC.2	TCTGCGCCGGTCACGCCC		
arpA (E)	ArpAgpE.f	GATTCCATCTTGTCAAAATATGCC	301	Lescat *et al*., 2013
	ArpAgpE.r	GAAAAGAAAAAGAATTCCCAAGAG		
trpA	trpBA.f	CGGCGATAAAGACATCTTCAC	489	Clermont *et al*., 2008
	trpBA.r	GCAACGCGGCCTGGCGGAAG		
**Primers used for amplification and sequencing following the Achtman seven-locus scheme**				
adk (locus size: 536 bp)	adkF	ATTCTGCTTGGCGCTCCGGG	975	Wirth *et al*., 2006
	adkR	CCGTCAACTTTCGCGTATTT		
fumC (locus size: 469 bp)	fumCF	TCACAGGTCGCCAGCGCTTC	806	
	fumCR	GTACGCAGCGAAAAAGATTC		
gyrB (locus size: 460 bp)	gyrBF	TCGGCGACACGGATGACGGC	911	
	gyrBR	ATCAGGCCTTCACGCGCATC		
icd (locus size: 518)	icdF	ATGGAAAGTAAAGTAGTTGTTCCGGCACA	878	
	icdR	GGACGCAGCAGGATCTGTT		
mdh (locus size: 452 bp)	mdhF	ATGAAAGTCGCAGTCCTCGGCGCTGCTGGCGG	932	
	mdhR	TTAACGAACTCCTGCCCCAGAGCGATATCTTTCTT		
purA (locus size: 478 bp)	purAF	CGCGCTGATGAAAGAGATGA	816	
	purAR	CATACGGTAAGCCACGCAGA		
recA (locus size: 510 bp)	recAR1	AGCGTGAAGGTAAAACCTGTG	780	
	recAF1	ACCTTTGTAGCTGTACCACG		
**Primers used for amplification and sequencing in the clonotyping method**				
fimH (locus size: 469 bp)	fimH-F	CACTCAGGGAACCATTCAGGCA	975	Weissman *et al*., 2012
	fimH-R	CTTATTGATAAACAAAAGTCAC		

Table S5. ST allele combinations found among the 65 diarrheagenic *mcr-1 E. coli* isolates: association with clonotypes and pathotypes

Phylo group	ST COMPLEX	[a]ST	*adk*	*fumC*	*gyrB*	*icd*	*mdh*	*purA*	*recA*	[b]Clonotypes (No. isolates and pathotype)
A	ST10 Cplx	10	10	11	4	8	8	8	2	11-23 (1 STEC)<br>**11-24** (14 ETEC; 5 STEC/ETEC; 4 aEPEC; 1 STEC)<br>11-45 (1 ETEC)<br>11-94 (2 ETEC)<br>11-0 (1 ETEC)
		48	6	11	4	8	8	8	2	11-54 (3 aEPEC)
		5786	10	11	4	8	8	8	405	**11-24** (5 ETEC)
		7367	6	685	4	8	8	8	2	685-54 (1 aEPEC)
	ST165 Cplx	100	10	27	5	10	12	9	2	27-0 (2 ETEC)
		301	78	27	5	10	12	8	2	27-54 (3 aEPEC)
		STNew1	78	27	5	10	8	8	2	27-54 (1 aEPEC)
	ST168 Cplx	93	6	11	4	10	7	8	6	11-27 (1 ETEC)
	ST398Cplx	398	64	7	1	1	8	8	6	7-171 (1 ETEC)
B1	ST156 Cplx	156	6	29	32	16	11	8	44	29-38 (1 ETEC)
	ST20 Cplx	20	6	4	3	18	7	7	6	4-25 (1 aEPEC)
	ST29 Cplx	29	6	4	12	16	9	7	7	**4-24** (7 aEPEC)<br>4-440 (2 aEPEC)
E	ST32 Cplx	1034	19	23	51	176	21	2	16	23-331 (1 aEPEC)
	-	1	4	2	2	4	4	4	4	2-54 (1 STEC)
	-	42	23	28	22	27	5	16	4	28-65 (3 ETEC)
	-	118	31	4	42	44	15	33	17	4-331 (1 ETEC)
	-	302	79	84	71	78	52	57	2	84-305 (1 aEPEC)
	-	4247	83	550	280	453	112	2	138	550-400 (1 ETEC)

[a] STNew1 (1 SLV of ST301 and 2 SLV of ST165) would be included into the ST165 Cplx (- = without assignation).
[b] Clonotype: *fumC-fimH* alleles; 0 = *fimH* negative by PCR; in blue, prevalent clonotypes.

Table S6. Polymorphic sites in the deduced FedA amino acid sequences of the 18 porcine *E. coli* isolated in this this study compared with those of Barth et al. (2011)

Isolate ID[a]	FedA	Amino acid sequence[b]																											
	Subtype	31	43	55	56	57	59	61	63	66	74	75	79	83	88	93	102	105	107	110	112	116	121	122	123	134	143	148	155	156
GQ325624	ac	S	I	T	V	T	N	T	I	P	T	T	A	N	I	Q	Q	V	R	Y	A	V	Q	P	A	N	E	T	Q	T
GQ325625	ac	S	I	T	A	T	N	T	R	P	T	A	A	N	I	Q	Q	V	N	Y	A	V	Q	L	A	S	E	T	Q	T
GQ325626	ac	S	I	T	A	T	N	T	R	P	T	A	A	N	I	Q	Q	V	N	Y	A	V	Q	L	A	S	E	T	Q	T
GQ325627	ac	S	I	T	V	T	N	T	S	H	A	T	A	N	I	Q	Q	V	R	Y	A	V	Q	P	G	S	E	I	Q	T
GQ325628	ac	S	I	T	A	T	N	T	S	P	T	A	A	N	I	Q	Q	V	N	Y	A	V	Q	L	A	S	E	T	Q	T
GQ325629	ac	S	I	T	A	T	N	T	S	P	T	A	A	N	I	Q	Q	V	N	Y	A	V	Q	L	A	S	E	T	Q	T
GQ325630	ac	S	V	T	V	T	N	T	S	P	A	A	A	N	I	Q	Q	V	N	Y	A	V	Q	S	A	S	E	T	Q	T
GQ325631	ac	S	V	T	V	T	N	T	S	P	A	A	A	N	I	Q	Q	V	N	Y	A	V	R	P	A	S	E	T	Q	T
GQ325632	ac	S	I	T	V	T	N	T	S	P	T	A	A	N	I	Q	Q	V	N	Y	A	V	Q	A	A	N	E	T	Q	T
GQ325633	New	N	I	S	V	T	N	V	G	P	A	T	S	S	V	Q	Q	V	N	Y	A	A	Q	M	V	S	E	T	Q	T
GQ325621	ab	N	I	T	V	A	S	A	R	P	N	V	A	T	I	Q	Q	V	N	Y	A	V	Q	-	G	S	D	T	Q	T
GQ325622	ab	N	I	T	V	A	S	T	S	P	N	V	A	T	I	Q	Q	V	N	Y	A	V	Q	-	G	S	D	T	Q	P
GQ325623	ab	N	I	T	V	A	S	A	R	P	N	V	A	T	I	Q	Q	V	N	Y	A	V	Q	-	G	S	E	T	Q	T
FV9821*	ac	S	I	T	V	T	N	T	I	P	T	T	A	N	I	Q	Q	V	R	Y	A	V	Q	P	A	N	E	T	Q	T
FV10406*	ac	S	I	T	V	T	N	T	S	P	T	T	A	N	I	Q	Q	V	R	Y	A	V	Q	P	A	N	E	T	Q	T
FV10466*	ac	S	I	T	V	T	N	N	V	P	A	T	A	N	I	Q	Q	V	R	H	F	V	Q	P	A	S	E	T	Q	T
FV11288*	ac	S	I	T	V	T	N	N	V	P	A	T	A	N	I	Q	Q	V	R	H	F	V	Q	P	A	S	E	T	Q	T
FV11519*	ac	S	I	T	V	T	N	T	I	P	T	T	A	N	I	Q	Q	V	R	Y	A	V	Q	P	A	N	E	T	Q	T
FV13237*	ac	S	I	T	V	T	N	N	V	P	A	T	A	N	I	Q	Q	V	R	H	F	V	Q	P	A	S	E	T	Q	T
FV13627*	ac	S	I	T	V	T	N	I	S	P	T	T	A	N	I	Q	Q	V	R	Y	A	V	Q	P	A	N	E	T	P	T
FV14101*	ac	S	I	T	V	T	N	I	S	P	T	T	A	N	I	Q	Q	V	R	Y	A	V	Q	P	A	N	E	T	Q	T
FV14146*	ac	S	I	T	V	T	N	A	S	P	T	T	A	N	I	Q	Q	V	R	Y	A	V	Q	P	A	N	E	T	Q	T
FV14450*	ac	S	I	T	V	T	N	I	S	P	T	T	A	N	I	Q	Q	V	R	Y	A	V	Q	P	A	N	E	T	Q	T
FV15900*	ac	S	I	T	V	T	N	T	I	P	A	T	A	N	I	K	Q	V	R	Y	A	V	Q	P	A	N	E	T	Q	T
FV15917*	ac	S	I	T	V	T	N	T	I	P	T	T	A	N	I	Q	Q	V	R	Y	A	V	Q	P	A	N	E	T	Q	T
FV17904*	ac	S	I	T	V	T	N	T	I	P	T	T	A	N	I	Q	Q	V	R	Y	A	V	Q	P	A	N	E	T	Q	T
FV18451*	ac	S	I	T	V	T	N	I	S	P	T	T	A	N	I	Q	Q	V	R	Y	A	V	Q	P	A	N	E	T	Q	T
FV18738*	ac	S	I	T	V	T	N	T	S	P	T	T	A	N	I	Q	L	V	R	H	F	V	Q	P	T	S	E	T	Q	T

FV18841*	ac	S	I	T	V	T	N	S	S	P	T	T	A	N	I	Q	Q	V	R	Y	A	V	Q	P	A	N	E	T	Q	T
FV18854*	ac	S	I	T	V	T	G	N	G	P	A	T	A	N	I	Q	K	A	R	H	F	V	Q	P	T	S	E	T	Q	T
FV19067*	ab	N	I	T	V	A	S	T	S	P	N	V	A	T	I	Q	Q	V	N	Y	A	V	Q	-	G	S	D	T	Q	P

[a] Isolates of the present study are identified with *.

[b] In red, the amino acid positions that define fimbrial subtype according to Bosworth et al. (1998). Shaded in grey the amino acid differences for the fimbrial subtype positions detected in this study compared with those from Barth et al. (2011).

S, serine; I, isoleucine; T, threonine; V, valine; N, asparagine, P, proline; A, alanine; Q, glutamine; R, arginine; Y, tyrosine; E, glutamic acid; G, glycine; H, histidine; L, leucine; K, lysine; F, phenylalanine; M, methionine; D, aspartic acid; -, amino acid missing.

Figure S1. Phylogenetic analysis of the deduced FedA amino acid sequences of the 18 porcine *E. coli* isolated in this study and those described by Barth et al. (2011)

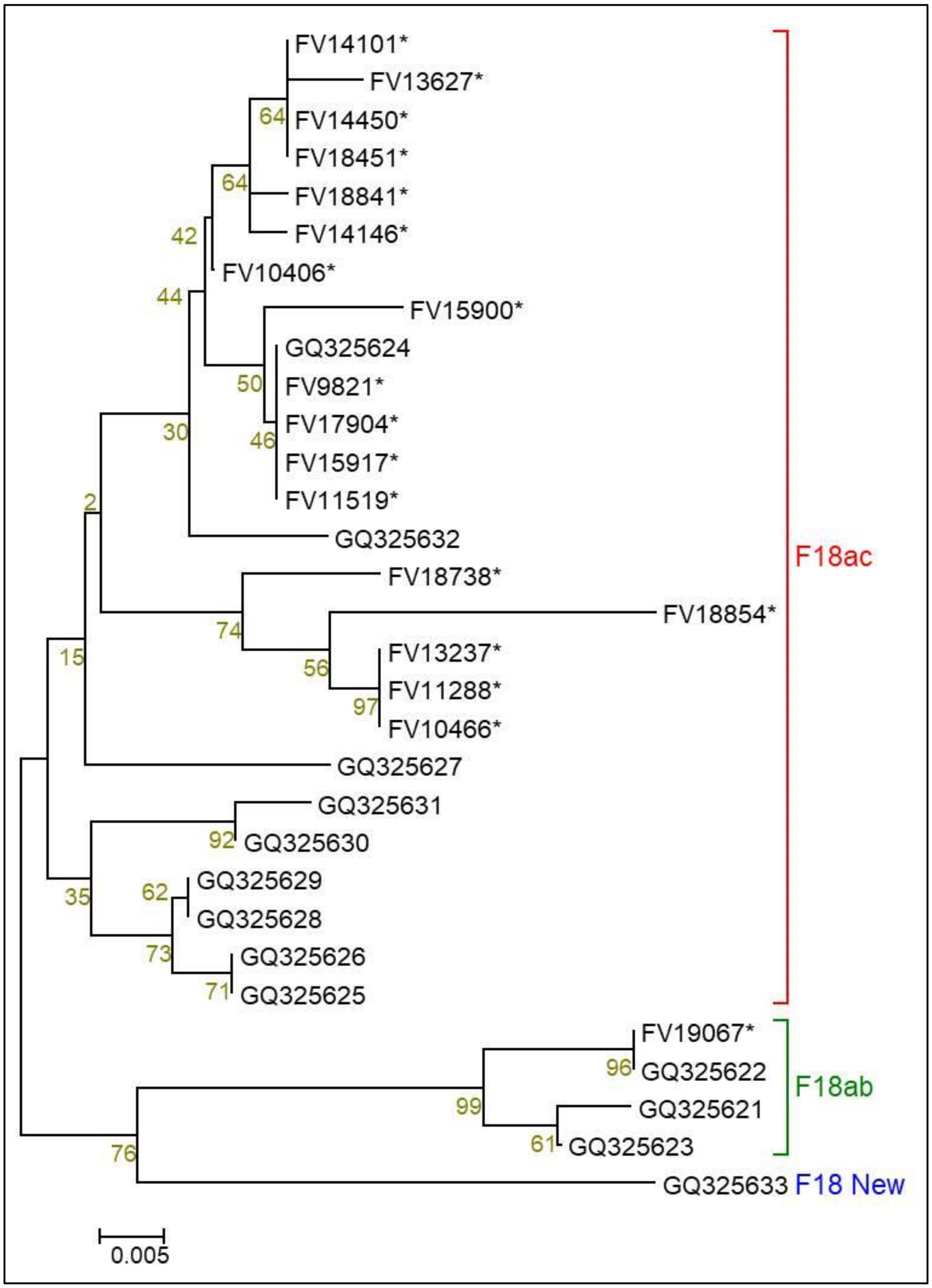

The phylogenetic tree was generated by the Neighbor-Joining method using MEGA6 (Tamura et al., 2013) and including all deduced FedA protein sequences detected in this study (identified with asterisk *) and those described by Barth et al. (2011) and designated with their respective GenBank accession numbers. Numbers on the tree indicate the bootstrap values calculated for 1,000 replicates.

Table S7. Nucleotide differences of the 8 *fimH* ST131 alleles of this study compared with those reported by Dahbi et al. (2014) in human isolates

*fim*H allele[a]	Nucleotide sequence[b]																									
	30	33	54	77	108	119	122	144	162	181	184	196	197	221	233	235	249	252	255	257	290	317	329	348	351	387	483
*fimH22**	A	C	C	C	G	C	A	C	T	G	T	G	G	C	A	G	T	C	T	C	C	C	C	G	A	A	T
fimH375	A	C	C	T	G	C	A	C	T	G	T	G	G	C	A	G	T	C	T	C	C	C	C	G	A	A	T
fimH376	A	C	C	C	G	C	A	C	T	A	T	G	G	C	A	G	T	C	T	C	C	C	C	G	A	A	T
*fimH332**	A	C	C	C	G	C	A	C	T	G	T	C	G	C	A	G	T	C	T	C	C	C	C	G	A	A	T
fimH324	A	C	C	C	G	C	A	C	T	G	T	T	G	C	A	G	T	C	T	C	C	C	C	G	A	A	T
*fimH326**	A	C	C	C	G	C	A	C	T	G	T	G	G	C	A	G	T	C	T	C	C	C	T	G	A	A	T
*fimH338**	A	C	C	C	G	C	A	C	T	G	G	G	G	C	A	G	T	C	T	C	C	C	C	G	A	A	T
fimH378	A	C	C	C	G	C	T	C	T	G	T	G	G	C	A	G	T	C	T	C	C	C	C	G	A	A	T
*fimH330**	A	C	C	C	G	C	A	C	T	G	T	G	T	C	A	G	T	C	T	C	C	C	C	G	A	A	T
fimH207	A	C	C	C	G	C	A	C	T	G	T	G	A	C	A	G	T	C	T	C	C	C	C	G	A	A	T
*fimH161**	A	C	C	C	G	C	A	C	T	G	T	G	G	C	A	G	T	C	T	C	C	T	C	G	A	A	T
*fimH336**	A	C	C	C	G	C	A	C	T	G	T	G	G	C	A	G	T	C	T	G	C	T	C	G	A	A	T
*fimH374**	A	C	C	C	G	C	A	C	T	G	T	A	G	T	A	G	T	C	T	C	C	C	C	G	A	A	T
fimH30	T	T	T	C	A	C	A	T	C	G	T	G	G	C	G	G	A	T	G	C	C	C	C	T	G	T	C
fimH381	T	T	T	C	A	G	A	T	C	G	T	G	G	C	G	A	A	T	G	C	T	C	C	T	G	T	C

[a] Alleles detected in the present study are indicated with asterisk * and underlined those from Dahbi et al. (2014). [b] Shaded in grey the nucleotidic differences in relation to the *fimH22* allele. A, adenine; C, cytosine; T, thymine; G, guanine.

Figure S2. Phylogenetic analysis of the 8 *fimH* ST131 alleles detected in this study (indicated with asterisk *) and those reported by Dahbi et al. (2014) in human isolates (underlined). The phylogenetic tree was generated by the Neighbor-Joining method using MEGA6 (Tamura et al., 2013); numbers on the tree indicate the bootstrap values calculated for 1,000 replicates.

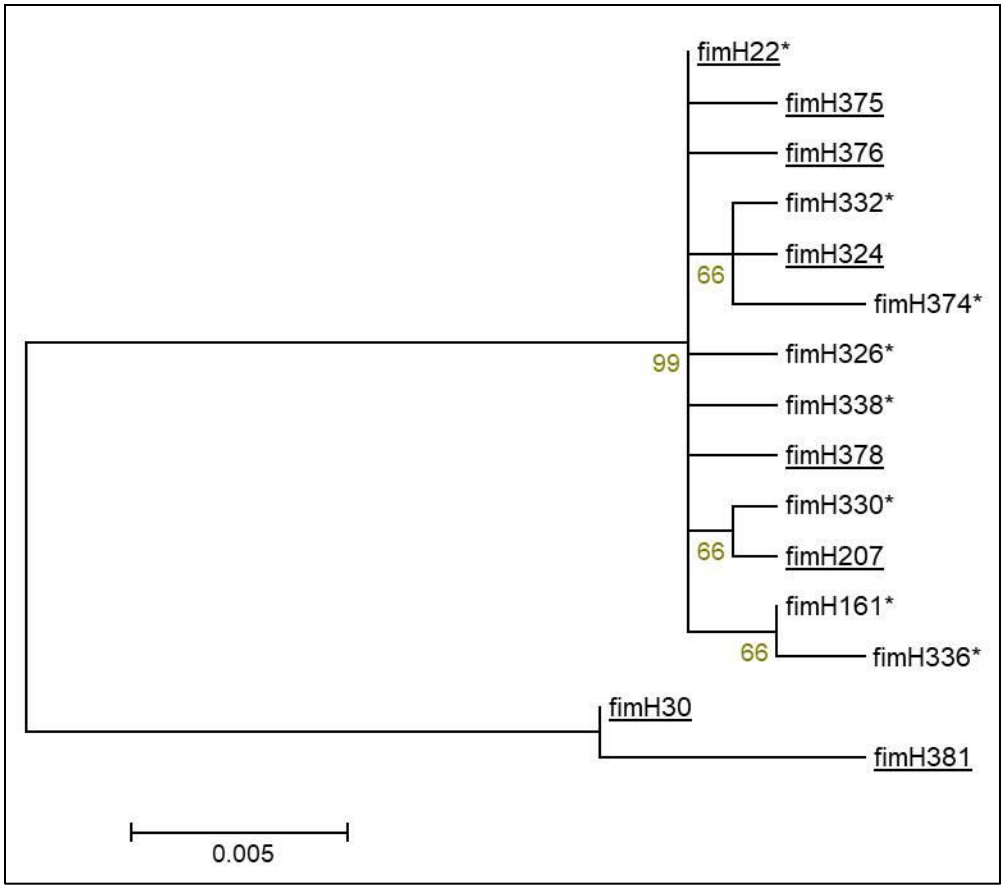

Table S8. Amino acid (aa) differences of the 8 *fimH* ST131 alleles of this study compared with those reported by Dahbi et al. (2014) in human isolates

fimH allele[a]	aa sequence[b]												
	26	40	41	61	62	66	74	78	79	86	97	106	110
*fimH22**	P	T	Q	G	S	G	T	N	G	T	S	A	T
fimH375	L	T	Q	G	S	G	T	N	G	T	S	A	T
fimH376	P	T	Q	S	S	G	T	N	G	T	S	A	T
*fimH332**	P	T	Q	G	S	R	T	N	G	T	S	A	T
fimH324	P	T	Q	G	S	C	T	N	G	T	S	A	T
*fimH326**	P	T	Q	G	S	G	T	N	G	T	S	A	M
*fimH338**	P	T	Q	G	A	G	T	N	G	T	S	A	T
fimH378	P	T	L	G	S	G	T	N	G	T	S	A	T
*fimH330**	P	T	Q	G	S	V	T	N	G	T	S	A	T
fimH207	P	T	Q	G	S	D	T	N	G	T	S	A	T
*fimH161**	P	T	Q	G	S	G	T	N	G	T	S	V	T
*fimH336**	P	T	Q	G	S	G	T	N	G	S	S	V	T
*fimH374**	P	T	Q	G	S	S	I	N	G	T	S	A	T
fimH30	P	T	Q	G	S	G	T	S	G	T	S	A	T
fimH381	P	R	Q	G	S	G	T	S	S	T	L	A	T

[a] Alleles detected in the present study are indicated with asterisk * and underlined those from Dahbi et al. (2014). [b] Shaded in grey the aa differences in relation to the *fimH22* protein. P, proline; L, leucine; T, threonine; R, arginine; Q, glutamine; S, serine; G, glycine; C, cysteine; R, arginine; D, aspartic acid; V, valine; I, isoleucine; N, asparagine; A, alanine; M, methionine.

Figure S3. Phylogenetic analysis of the deduced FimH ST131 amino acid sequences detected in this study (indicated with asterisk *) and those reported by Dahbi et al. (2014) in human isolates (underlined). The phylogenetic tree was generated by the Neighbor-Joining method using MEGA6 (Tamura et al., 2013); numbers on the tree indicate the bootstrap values calculated for 1,000 replicates.

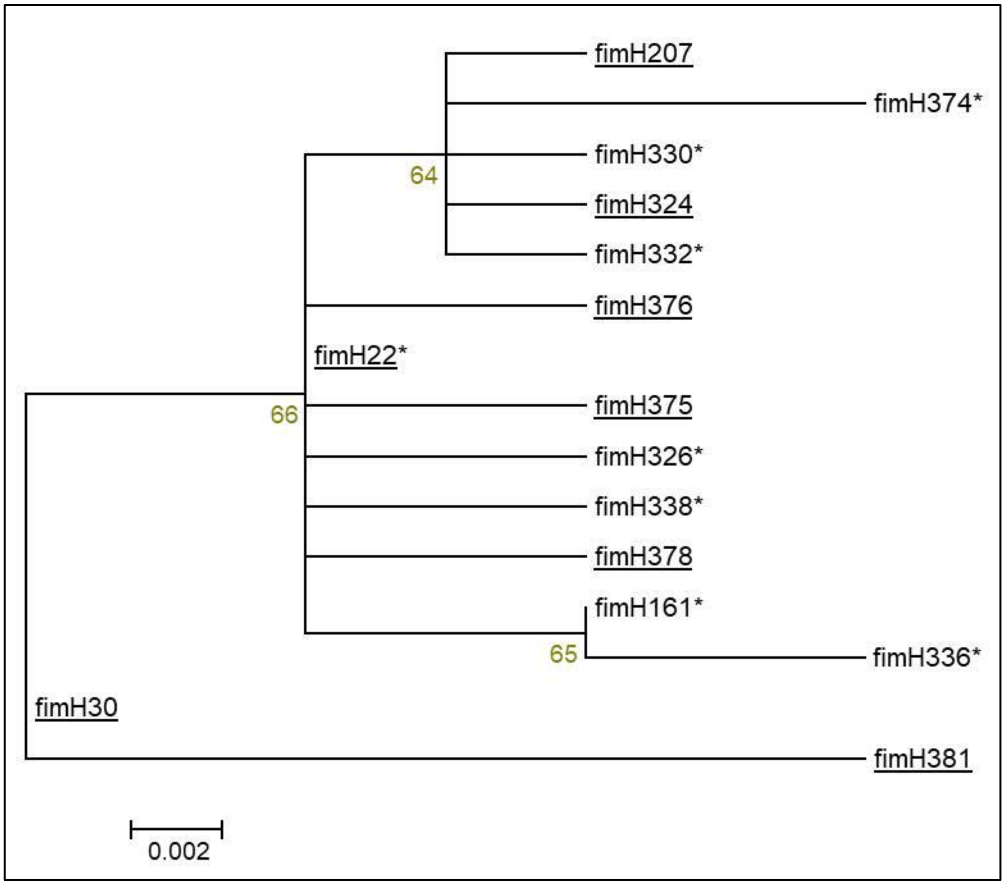

References

Alonso, C.A., Mora, A., Díaz, D., Blanco, M., González-Barrio, D., Ruiz-Fons, F., Simón, C., Blanco, J., Torres, C. (2017). Occurrence and characterization of *stx* and/or *eae*-positive *Escherichia coli* isolated from wildlife, including a typical EPEC strain from a wild boar. *Vet. Microbiol.* 207, 69-73.

Barth, S., Schwanitz, A., Bauerfeind, R. (2011). Polymerase chain reaction-based method for the typing of F18 fimbriae and distribution of F18 fimbrial subtypes among porcine Shiga toxin-encoding *Escherichia coli* in Germany. *J. Vet. Diagn. Invest.* 23, 454-464.

Bauer, R.J., Zhang, L., Foxman, B., Siitonen, A., Jantunen, M.E., Saxen, H., Marrs, C.F. (2002). Molecular epidemiology of 3 putative virulence genes for *Escherichia coli* urinary tract infection-*usp*, *iha*, and *iroN*(*E. coli*). *J. Infect. Dis.* 185, 1521-1524.

Bennett, J. (2003). Classical enteropathogenic *Escherichia coli* or atypical strains? Examination of shigatoxin negative, *eaeA* positive isolates received in the Enteric Reference Laboratory in 2000. *New Zealand Journal of Medical Laboratory Science* 57, 2-7.

Blanco, M., Blanco, J.E., Gonzalez, E.A., Mora, A., Jansen, W., Gomes, T.A., Zerbini, L.F., Yano, T., de Castro, A.F., Blanco, J. (1997). Genes coding for enterotoxins and verotoxins in porcine *Escherichia coli* strains belonging to different O:K:H serotypes: relationship with toxic phenotypes. *J. Clin. Microbiol.* 35, 2958-2963.

Blanco, M., Lazo, L., Blanco, J.E., Dahbi, G., Mora, A., López, C., González, E.A., Blanco, J. (2006). Serotypes, virulence genes, and PFGE patterns of enteropathogenic *Escherichia coli* isolated from Cuban pigs with diarrhea. *Int. Microbiol.* 9, 53-60.

Borowiak, M., Fischer, J., Hammerl, J.A., Hendriksen, R.S., Szabo, I., Malorny, B. (2017). Identification of a novel transposon-associated phosphoethanolamine transferase gene, *mcr-5*, conferring colistin resistance in d-tartrate fermenting *Salmonella enterica* subsp. *enterica* serovar Paratyphi B. *J. Antimicrob. Chemother.* 72, 3317-3324.

Bosworth, B.T., Dean-Nystrom, E.A., Casey, T.A., Neibergs, H.L. (1998). Differentiation of F18ab$^+$ from F18ac$^+$ *Escherichia coli* by single-strand conformational polymorphism analysis of the major fimbrial subunit gene (*fedA*). *Clin. Diagn. Lab. Immunol.*5, 299-302.

Carattoli, A., Villa, L., Feudi, C., Curcio, L., Orsini, S., Luppi, A., Pezzotti, G., Magistrali, C.F. (2017). Novel plasmid-mediated colistin resistance *mcr-4* gene in *Salmonella* and *Escherichia coli*, Italy 2013, Spain and Belgium, 2015 to 2016. *EuroSurveill*. 22.

Clermont, O., Bonacorsi, S., and Bingen, E. (2000). Rapid and simple determination of *Escherichia coli* phylogenetic group. *Appl Environ Microbiol*. 66, 4555-4558. doi: 10.1093/jac/dkp194.

Clermont, O., Lavollay, M., Vimont, S., Deschamps, C., Forestier, C., Branger, C., et al. (2008). The CTX-M-15-producing *Escherichia coli* diffusing clone belongs to a highly virulent B2 phylogenetic subgroup. *J. Antimicrob. Chemother.* 61, 1024-1028. doi: 10.1093/jac/dkn084.

Clermont, O., Christenson, J.K., Denamur, E., and Gordon, D.M. (2013). The Clermont *Escherichia coli* phylo-typing method revisited: improvement of specificity and detection of new phylo-groups. *Environ. Microbiol. Rep.* 5, 58-65. doi: 10.1111/1758-2229.12019.

Dahbi, G., Mora, A., Mamani, R., López, C., Alonso, M.P., Marzoa, J., Blanco, M., Herrera, A., Viso, S., García-Garrote, F., Tchesnokova, V., Billig, M., de la Cruz, F., de Toro, M., González-López, J.J., Prats, G., Chaves, F., Martínez-Martínez, L., López-Cerezo, L., Denamur, E., Blanco, J. (2014). Molecular epidemiology and virulence of *Escherichia coli* O16:H5-ST131: comparison with H30 and H30-Rx subclones of O25b:H4-ST131. *Int. J. Med. Microbiol.* 304, 1247-1257.

Dozois, C.M., Dho-Moulin, M., Brée, A., Fairbrother, J.M., Desautels, C., Curtiss, R., 3rd. (2000). Relationship between the Tsh autotransporter and pathogenicity of avian *Escherichia coli* and localization and analysis of the Tsh genetic region. *Infect. Immun.* 68, 4145-4154.

Durso, L.M., Bono, J.L., Keen, J.E. (2005). Molecular serotyping of *Escherichia coli* O26:H11. *Appl. Environ. Microbiol.* 71, 4941-4944.

Franklin, M.A., Francis, D.H., Baker, D., Mathew, A.G. (1996). A PCR-based method of detection and differentiation of K88+ adhesive *Escherichia coli*. *J. Vet. Diagn. Invest.* 8, 460-463.

Gannon, V.P., D'Souza, S., Graham, T., King, R.K., Rahn, K., Read, S. (1997). Use of the flagellar H7 gene as a target in multiplex PCR assays and improved specificity in identification of enterohemorrhagic *Escherichia coli* strains. *J. Clin. Microbiol.* 35, 656-662.

Imberechts, H., De Greve, H., Schlicker, C., Bouchet, H., Pohl, P., Charlier, G., Bertschinger, H., Wild, P., Vandekerckhove, J., Van Damme, J., et al. (1992). Characterization of F107 fimbriae of *Escherichia coli* 107/86, which causes edema disease in pigs, and nucleotide sequence of the F107 major fimbrial subunit gene, *fedA*. *Infect. Immun.* 60, 1963-1971.

Johnson, J.R., Gajewski, A., Lesse, A.J., Russo, T.A. (2003). Extraintestinal pathogenic *Escherichia coli* as a cause of invasive nonurinary infections. *J. Clin. Microbiol.* 41, 5798-5802.

Johnson, J.R., O'Bryan, T.T. (2004). Detection of the *Escherichia coli* group 2 polysaccharide capsule synthesis Gene kpsM by a rapid and specific PCR-based assay. *J. Clin. Microbiol.* 42, 1773-1776.

Johnson, J.R., Porter, S., Johnston, B., Kuskowski, M.A., Spurbeck, R.R., Mobley, H.L., Williamson, D.A. (2015). Host Characteristics and Bacterial Traits Predict Experimental Virulence for *Escherichia coli* Bloodstream Isolates From Patients With Urosepsis. *Open. Forum Infect. Dis*. 2, ofv083.

Johnson, J.R., Russo, T.A., Tarr, P.I., Carlino, U., Bilge, S.S., Vary, J.C., Jr., Stell, A.L. (2000). Molecular epidemiological and phylogenetic associations of two novel putative virulence genes, *iha* and *iroN*(*E. coli*), among *Escherichia coli* isolates from patients with urosepsis. *Infect. Immun.* 68, 3040-3047.

Johnson, J.R., Stapleton, A.E., Russo, T.A., Scheutz, F., Brown, J.J., Maslow, J.N. (1997). Characteristics and prevalence within serogroup O4 of a J96-like clonal group of

uropathogenic *Escherichia coli* O4:H5 containing the class I and class III alleles of *papG*. *Infect. Immun.* 65, 2153-2159.

Johnson, J.R., Stell, A.L. (2000). Extended virulence genotypes of *Escherichia coli* strains from patients with urosepsis in relation to phylogeny and host compromise. *J. Infect. Dis.* 181, 261-272.

Le Bouguenec, C., Archambaud, M., Labigne, A. (1992). Rapid and specific detection of the *pap*, *afa*, and *sfa* adhesin-encoding operons in uropathogenic *Escherichia coli* strains by polymerase chain reaction. *J. Clin. Microbiol.* 30, 1189-1193.

Lescat, M., Clermont, O., Woerther, P.L., Glodt, J., Dion, S., Skurnik, D., *et al*. (2013). Commensal *Escherichia coli* strains in Guiana reveal a high genetic diversity with host-dependant population structure. *Environ Microbiol Rep*. 5, 49-57. doi: 10.1111/j.1758-2229.2012.00374.x.

Liu, Y.Y., Wang, Y., Walsh, T.R., Yi, L.X., Zhang, R., Spencer, J., Doi, Y., Tian, G., Dong, B., Huang, X., Yu, L.F., Gu, D., Ren, H., Chen, X., Lv, L., He, D., Zhou, H., Liang, Z., Liu, J.H., Shen, J. (2016). Emergence of plasmid-mediated colistin resistance mechanism MCR-1 in animals and human beings in China: a microbiological and molecular biological study. *Lancet Infect. Dis.* 16, 161-168.

Marc, D., Dho-Moulin, M. (1996). Analysis of the *fim* cluster of an avian O2 strain of *Escherichia coli*: serogroup-specific sites within *fimA* and nucleotide sequence of *fimI*. *J. Med. Microbiol.* 44, 444-452.

Mora, A., García-Pena, F.J., Alonso, M.P., Pedraza-Diaz, S., Ortega-Mora, L.M., Garcia-Parraga, D., López, C., Viso, S., Dahbi, G., Marzoa, J., Sergeant, M.J., García, V., Blanco, J. (2018). Impact of human-associated *Escherichia coli* clonal groups in Antarctic pinnipeds: presence of ST73, ST95, ST141 and ST131. *Sci. Rep.* 8, 4678.

Mora, A., Herrrera, A., Lopez, C., Dahbi, G., Mamani, R., Pita, J.M., Alonso, M.P., Llovo, J., Bernardez, M.I., Blanco, J.E., Blanco, M., Blanco, J. (2011). Characteristics of the Shiga-toxin-producing enteroaggregative *Escherichia coli* O104:H4 German outbreak strain and of STEC strains isolated in Spain. *Int. Microbiol.* 14, 121-141.

Mora, A., López, C., Dhabi, G., López-Beceiro, A.M., Fidalgo, L.E., Díaz, E.A., Martínez-Carrasco, C., Mamani, R., Herrera, A., Blanco, J.E., Blanco, M., Blanco, J. (2012). Seropathotypes, Phylogroups, Stx subtypes, and intimin types of wildlife-carried, shiga toxin-producing *Escherichia coli* strains with the same characteristics as human-pathogenic isolates. *Appl. Environ. Microbiol.* 78, 2578-2585.

Mora, A., Viso, S., López, C., Alonso, M.P., García-Garrote, F., Dabhi, G., Mamani, R., Herrera, A., Marzoa, J., Blanco, M., Blanco, J.E., Moulin-Schouleur, M., Schouler, C., Blanco, J. (2013). Poultry as reservoir for extraintestinal pathogenic *Escherichia coli* O45:K1:H7-B2-ST95 in humans. *Vet. Microbiol.* 167, 506-512.

Moulin-Schouleur, M., Schouler, C., Tailliez, P., Kao, M.R., Brée, A., Germon, P., Oswald, E., Mainil, J., Blanco, M., Blanco, J. (2006). Common virulence factors and genetic relationships between O18:K1:H7 *Escherichia coli* isolates of human and avian origin. *J. Clin. Microbiol.* 44, 3484-3492.

Penteado, A.S., Ugrinovich, L.A., Blanco, J., Blanco, M., Blanco, J.E., Mora, A., Andrade, J.R., Correa, S.S., Pestana de Castro, A.F. (2002). Serobiotypes and virulence genes of *Escherichia coli* strains isolated from diarrheic and healthy rabbits in Brazil. *Vet. Microbiol.* 89, 41-51.

Rasheed, J.K., Jay, C., Metchock, B., Berkowitz, F., Weigel, L., Crellin, J., Steward, C., Hill, B., Medeiros, A.A., Tenover, F.C. (1997). Evolution of extended-spectrum beta-lactam resistance (SHV-8) in a strain of *Escherichia coli* during multiple episodes of bacteremia. *Antimicrob. Agents Chemother.* 41, 647-653.

Saladin, M., Cao, V.T., Lambert, T., Donay, J.L., Herrmann, J.L., Ould-Hocine, Z., Verdet, C., Delisle, F., Philippon, A., Arlet, G. (2002). Diversity of CTX-M beta-lactamases and their promoter regions from Enterobacteriaceae isolated in three Parisian hospitals. *FEMS Microbiol. Lett.* 209, 161-168.

Scheutz, F., Teel, L.D., Beutin, L., Pierard, D., Buvens, G., Karch, H., Mellmann, A., Caprioli, A., Tozzoli, R., Morabito, S., Strockbine, N.A., Melton-Celsa, A.R., Sanchez, M., Persson, S., O'Brien, A.D. (2012). Multicenter evaluation of a sequence-based protocol for subtyping Shiga toxins and standardizing Stx nomenclature. *J. Clin. Microbiol.* 50, 2951-2963.

Schultsz, C., Pool, G.J., van Ketel, R., de Wever, B., Speelman, P., Dankert, J. (1994). Detection of enterotoxigenic *Escherichia coli* in stool samples by using nonradioactively labeled oligonucleotide DNA probes and PCR. *J. Clin. Microbiol.* 32, 2393-2397.

Simarro, E., Navarro, F., Ruiz, J., Miró, E., Gómez, J., Mirelis, B. (2000). *Salmonella enterica* serovar virchow with CTX-M-like beta-lactamase in Spain. *J. Clin. Microbiol.* 38, 4676-4678.

Spurbeck, R.R., Dinh, P.C., Jr., Walk, S.T., Stapleton, A.E., Hooton, T.M., Nolan, L.K., Kim, K.S., Johnson, J.R., Mobley, H.L. (2012). *Escherichia coli* isolates that carry *vat*, *fyuA*, *chuA*, and *ycfV* efficiently colonize the urinary tract. *Infect. Immun.* 80, 4115-4122.

Tamura, K., Stecher, G., Peterson, D., Filipski, A., Kumar, S. (2013). MEGA6: Molecular Evolutionary Genetics Analysis version 6.0. *Mol. Biol. Evol.* 30, 2725-2729.

Tóth, I., Hérault, F., Beutin, L., Oswald, E. (2003). Production of cytolethal distending toxins by pathogenic *Escherichia coli* strains isolated from human and animal sources: establishment of the existence of a new cdt variant (Type IV). *J. Clin. Microbiol.* 41, 4285-4291.

Weissman, S.J., Johnson, J.R., Tchesnokova, V., Billig, M., Dykhuizen, D., Riddell, K., et al. (2012). High-resolution two-locus clonal typing of extraintestinal pathogenic *Escherichia coli*. *Appl. Environ. Microbiol.* 78, 1353-1360. doi: 10.1128/AEM.06663-11.

Wirth, T., Falush, D., Lan, R., Colles, F., Mensa, P., Wieler, L.H., et al. (2006). Sex and virulence in *Escherichia coli*: an evolutionary perspective. *Mol. Microbiol.* 60, 1136-1151. doi: 10.1111/j.1365-2958.2006.05172.x.

Xavier, B.B., Lammens, C., Ruhal, R., Kumar-Singh, S., Butaye, P., Goossens, H., Malhotra-Kumar, S. (2016). Identification of a novel plasmid-mediated colistin-resistance gene, *mcr-2*, in *Escherichia coli*, Belgium, June 2016. *EuroSurveill.* 21.

Yamamoto, S., Terai, A., Yuri, K., Kurazono, H., Takeda, Y., Yoshida, O. (1995). Detection of urovirulence factors in *Escherichia coli* by multiplex polymerase chain reaction. *FEMS Immunol. Med. Microbiol.* 12, 85-90.

Yin, W., Li, H., Shen, Y., Liu, Z., Wang, S., Shen, Z., Zhang, R., Walsh, T.R., Shen, J., Wang, Y. (2017). Novel Plasmid-Mediated Colistin Resistance Gene *mcr-3* in *Escherichia coli*. *MBio* 8.
